# A systematic scoping review of clinical indications for induction of labour

**DOI:** 10.1371/journal.pone.0228196

**Published:** 2020-01-29

**Authors:** Dominiek Coates, Angela Makris, Christine Catling, Amanda Henry, Vanessa Scarf, Nicole Watts, Deborah Fox, Purshaiyna Thirukumar, Vincent Wong, Hamish Russell, Caroline Homer

**Affiliations:** 1 Centre for Midwifery and Child and Family Health, Faculty of Health, University of Technology Sydney, Australia; 2 Department of Medicine, Western Sydney University, Sydney, Australia; 3 Women’s Health Initiative Translational Unit (WHITU), Liverpool Hospital, Liverpool, Australia; 4 School of Women’s and Children’s Health, UNSW Medicine, University of New South Wales, Sydney, Australia; 5 Department of Women’s and Children’s Health, St George Hospital, Sydney, Australia; 6 The George Institute for Global Health, UNSW Medicine, Sydney, Australia; 7 Liverpool Diabetes Collaborative Research Unit, Ingham Institute of Applied Research Science, University of New South Wales, Liverpool, Australia; 8 South Western Sydney Local Health District, Sydney, Australia; 9 Maternal and Child Health Program, Burnet Institute, Victoria, Australia; Ospedale dei Bambini Vittore Buzzi, ITALY

## Abstract

**Background:**

The proportion of women undergoing induction of labour (IOL) has risen in recent decades, with significant variation within countries and between hospitals. The aim of this study was to review research supporting indications for IOL and determine which indications are supported by evidence and where knowledge gaps exist.

**Methods:**

A systematic scoping review of quantitative studies of common indications for IOL. For each indication, we included systematic reviews/meta-analyses, randomised controlled trials (RCTs), cohort studies and case control studies that compared maternal and neonatal outcomes for different modes or timing of birth. Studies were identified via the databases PubMed, Maternity and Infant Care, CINAHL, EMBASE, and ClinicalTrials.gov from between April 2008 and November 2019, and also from reference lists of included studies. We identified 2554 abstracts and reviewed 300 full text articles. The quality of included studies was assessed using the RoB 2.0, the ROBINS-I and the ROBIN tool.

**Results:**

68 studies were included which related to post-term pregnancy (15), hypertension/pre-eclampsia (15), diabetes (9), prelabour rupture of membranes (5), twin pregnancy (5), suspected fetal compromise (4), maternal elevated body mass index (BMI) (4), intrahepatic cholestasis of pregnancy (3), suspected macrosomia (3), fetal gastroschisis (2), maternal age (2), and maternal cardiac disease (1). Available evidence supports IOL for women with post-term pregnancy, although the evidence is weak regarding the timing (41 versus 42 weeks), and for women with hypertension/preeclampsia in terms of improved maternal outcomes. For women with preterm premature rupture of membranes (24–37 weeks), high-quality evidence supports expectant management rather than IOL/early birth. Evidence is weakly supportive for IOL in women with term rupture of membranes. For all other indications, there were conflicting findings and/or insufficient power to provide definitive evidence.

**Conclusions:**

While for some indications, IOL is clearly recommended, a number of common indications for IOL do not have strong supporting evidence. Overall, few RCTs have evaluated the various indications for IOL. For conditions where clinical equipoise regarding timing of birth may still exist, such as suspected macrosomia and elevated BMI, researchers and funding agencies should prioritise studies of sufficient power that can provide quality evidence to guide care in these situations.

## Introduction

Induction of labour (IOL) has been on the rise over recent decades [1, 2], with significant variation within countries and between hospitals [1, 3, 4]. In Australia, the IOL rate rose from 27.3% in 2012 to 31.1% in 2016 [1].

IOL is generally undertaken with the aim of decreasing maternal and/or fetal morbidity or mortality i.e. when the risks of continuing the pregnancy to either mother or fetus are considered greater than the risks associated with planned birth [5]. For example, women are commonly induced for post-term pregnancy to reduce the risk of stillbirth [6]. Women with premature rupture of membranes are induced to decrease incidence of maternal sepsis and neonatal infection secondary to chorioamnionitis [7], women with preeclampsia to reduce the risk of stillbirth and severe maternal morbidity (renal failure, liver failure, coagulopathy, pulmonary oedema, eclamptic seizures) [8] and women with diabetes are induced to minimise macrosomia-associated birth complications and stillbirth risk [9].

While some indications for IOL are supported by high level evidence, others are not [10]. A systematic review of the evidence of indications by Mozurkewich et al. [10] conducted in 2008 found that the evidence at the time was insufficient to support IOL for women with common indications such as diabetes, twin gestation, suspected fetal macrosomia and oligohydramnios. The review called for further research to obtain a clearer picture of the risks and benefits associated with IOL [10].

In the 10 years since the initial review by Mozurkewich et al. [10], there continues to be debate about the acceptable use of IOL. There is no agreed external standard [11], and clinical guidelines vary considerably, both nationally and internationally [12–17]. The recent ARRIVE trial [18], which compared outcomes for low risk nulliparous women associated with IOL at 39 weeks (between 39+0 and 39+4) versus expectant management, seems to have further divided the maternity community in relation to IOL timing [19]. While this trial did not find any differences between the two groups for its primary outcome, that is, a composite of perinatal death and severe neonatal complications, it did find that IOL was associated with a reduction in caesarean section (CS) rate by 4%. This is at odds with some population studies that show that IOL is associated with a rise in CS rate [20]. Regardless of whether IOL is associated with a rise or reduction in CS rates, it is associated with increasing rates of early term birth [21], and its negative impact on child development [22]. Furthermore, IOL is often associated with a less positive birth experience for women compared to spontaneous onset of labour [23–25]. As such, the circumstances in which to offer a woman an IOL should be informed by the best available evidence.

The aim of this scoping review was to map the evidence in relation to indications for IOL and determine which are supported by evidence and where knowledge gaps exist. By building on the review by Mozurkewich et al. [10], this study presents a comprehensive overview of the available evidence to date.

## Method

We undertook a systematic scoping review [26–29], informed by the method used by Mozurkewich et al. [10], and following the PRISMA-ScR reporting guidelines for systematic reviews as outlined in our protocol developed before the review commenced. The aim of a scoping review is to map the literature relevant to a broad research topic to gain insight into the nature of the evidence and identify research gaps [26, 27, 29].

### Inclusion and exclusion criteria

We included quantitative studies that explored common indications for IOL, specifically: post-term pregnancy, premature rupture of membranes (PROM), twin pregnancy, antepartum haemorrhage, chorioamnionitis (including suspected), cholestasis of pregnancy, alloimmune disease or Rh disease, intrauterine growth restriction (IUGR), fetal distress, oligohydramnios, fetal gastroschisis, fetal macrosomia, fetal death, chronic/gestational hypertension, preeclampsia, diabetes, maternal age, elevated maternal body mass index (BMI), and other more uncommon maternal obstetric or medical indication (e.g. maternal cardiac disease, maternal melanoma, breast cancer, history of fast labour).

For each indication, we included systematic reviews/meta-analyses, randomised controlled trials (RCTs), prospective and retrospective cohort studies, and case control studies that compared maternal and neonatal outcomes for different modes or timing of birth i.e. IOL versus expectant management (EM); IOL versus immediate birth by caesarean section (CS); IOL at different time points (e.g. at 41 versus 42 weeks); and EM versus expedited birth.

To be included in the review, studies had to report on one or more of the following outcomes of interest: mode of birth, maternal morbidity, and fetal or neonatal morbidity and mortality. Following Mozurkewich et al. [10], maternal morbidity was defined as chorioamnionitis, endometritis, severe perineal trauma, blood transfusion, emergency CS or prolonged hospitalisation. Neonatal morbidity was defined as admission to a neonatal intensive care unit (NICU), 5-minute Apgar score <7, respiratory distress syndrome (RDS), shoulder dystocia, birth injury (as defined by the authors), meningitis, pneumonia, hypoxic ischaemic encephalopathy, meconium aspiration syndrome, or sepsis.

Studies were excluded if they were reported on in a systematic review or meta-analysis already included (as not to double count), where full text was not available or accessible, or if not published in English. Studies that compared different methods of IOL or evaluated IOL outcomes in the absence of a medical indication (e.g. maternal choice, routine IOL at 39 weeks) were also excluded.

### Search strategy

To identify studies for inclusion we searched the databases PubMed (including Cochrane Library), Maternity and Infant Care (OVID), CINAHL, EMBASE and ClinicalTrials.gov from April 2008 to April 2018, which was then updated with an additional search until November 2019 to ensure the review was up to date at the time of publication. The databases were searched using the terms ‘labour induction’, ‘induction’ and ‘induction of labour’ in combination with the indications listed above. Following this we examined the reference lists of included articles for further studies. We also included studies that met our inclusion criteria previously included in the review by Mozurkewich et al. [10], which covered the period from January 1980 to April 2008 and used a comprehensive search strategy. This approach means that our review presents a comprehensive overview of the evidence from 1980 to date.

### Data collection and extraction

**Fig 1:**
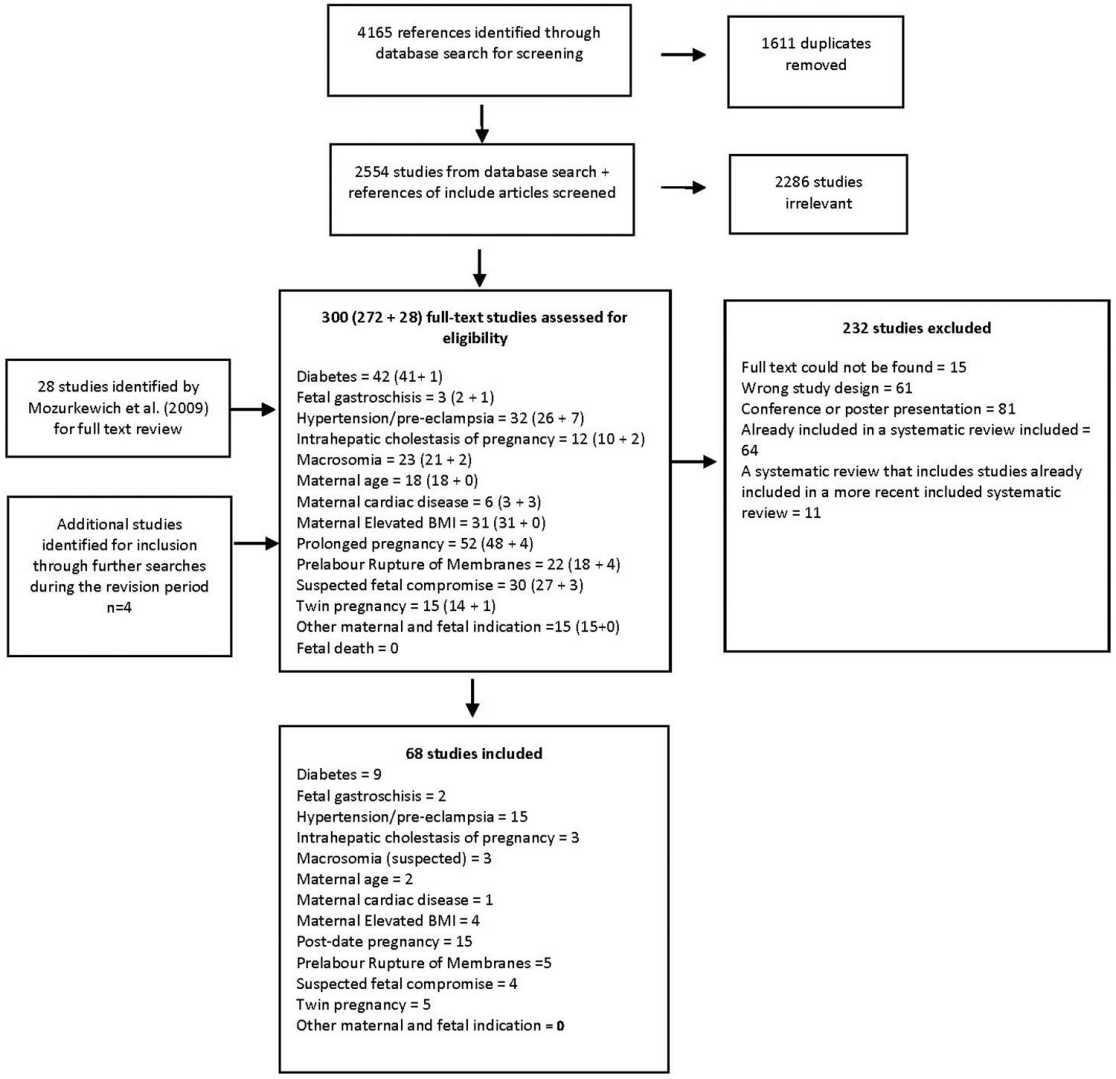
Flow of papers through review.

All articles were screened for eligibility against the review criteria by reading the title and abstract by one reviewer (first author). All full text articles were reviewed by two authors to determine suitability for inclusion (See Fig 1). For each of the included studies, data were extracted by two reviewers using a purposely designed template that followed the PICO framework (method, population, intervention and comparator, outcomes) [30]. The quality of included studies was assessed by two reviewers independently, using the Cochrane the Risk of Bias in Randomised Trials Tool (RoB 2.0 tool) for RCTs [31], the Risk Of Bias In Non-randomised Studies of Interventions tool (ROBINS-I) for non-randomised studies [32] and the ROBIN tool [33] for systematic reviews.

## Results

Our search identified 2554 papers for screening, of which 272 articles were identified for full text review. We also reviewed the 34 studies identified by Mozurkewich et al. [10], of which 28 were included for full text review (we could not access full text for the remaining six). In total, 300 full text studies were reviewed, of which 68 were included (see Tables 1 and 2). Only six of the studies included in the review by Mozurkewich et al. [10] were included here, as the remaining were studies already included in more recent systematic reviews, or were systematic reviews that had been superseded by more recent reviews.

**Table 1:** Included randomised controlled trials and meta-analyses of trials.

Author, Publication year	Indication	Study design	Country and Setting	Aim and Participants	Main Findings	Risk of bias
Gurung et al. (2013) [163]	Cholestasis	A Cochrane review of randomised and quasi randomised controlled trials	UK. While this review identified 21 RCTs for inclusion, only one RCT is relevant to our review and compared outcomes for early term delivery versus EM (the PITCH 2012 trial in the UK) and the findings of this study are reported here (N = 63)	N = 63 To evaluate the effectiveness and safety of interventions in women with cholestasis of pregnancy. Includes one RCT that compared outcomes for early term birth (n = 30) (IOL between 37+0 and 37+6) versus EM (n = 33).	There were no stillbirths or neonatal deaths in either group and no significant differences in CS (RR 0.68), passage of meconium-stained liquor (RR 0.55) or admission to NICU (RR 0.55).	Low
Boulvain et al. (2009) [145]	Diabetes (either type I or type II), or GDM	Cochrane review of RCTs	1 RCT included. Setting not specified in Cochrane review.	N = 200 To compare outcomes for IOL (n = 100) versus EM (n = 100) at term (≥38 weeks) for diabetic (either type I or type II, or GDM) pregnant women treated with insulin. Women with other complications were excluded.	No significant differences between the two groups in terms of CS (RR 0.81), the risk of macrosomia was reduced in the IOL group (RR 0.56) and three cases of mild shoulder dystocia were reported in the EM group. No other perinatal morbidity was reported.	Low
Biesty et al. (2018) [9]	Diabetes—GDM	Cochrane review of RCTs	1 RCT included. Multi-centre study conducted between 2010 and 2014, with teaching hospitals from Italy, Slovenia and Israel.	N = 425 To evaluate maternal and perinatal outcomes after IOL (n = 214) versus EM (n = 211) in pregnant women with GDM at term (included trial enrolled women at 38–39 weeks, excluded if estimated fetal weight over 4kg). Women with other complications (including diabetes type I or II) and previous CS were excluded.	No significant difference between the two groups in terms of CS rates (RR 1.06; 12.6% in the IOL group versus 11.7% in the expectant group), or other maternal or neonatal outcomes.	Low
Sutton et al. (2014) [146]	Diabetes—GDM	Secondary analysis of an RCT that compared different treatments for mild GDM.	Hospitals in North America that are members of the NICHD Maternal-Fetal Medicine Units Network	N = 679 (of the original 958 women) To compare CS rates associated with IOL (n = 220) versus EM (n = 459).	IOL was not associated with increased rates of CS at 37, 38, or 39 weeks, but was associated with a 3-fold increase in CS rates at 40 weeks and beyond.	Low
Grant et al. (2013) [182]	Gastrochisis	A Cochrane review of RCTs; studies with quasi randomised design or cross-over design were excluded	UK. Single centre RCT conducted between May 1995 and September 1999	N = 42 To assess the effects of planned preterm birth (< 37 weeks) for fetal gastroschisis by comparing outcomes for IOL at 36 weeks (n = 21) and spontaneous onset of labour (n = 21)	There was no significant benefit or adverse effect associated with elective preterm birth, but the included trial was underpowered to detect clinically important outcome differences.	Low
Amorim et al. (2017) [120]	Hypertension—Severe preeclampsia	Cochrane review of RCTs; quasi RCTs or studies with cross-over design were excluded	No studies identified for inclusion.	To compare planned CS versus IOL for severe preeclampsia	No studies identified for inclusion.	No included studies
Chappell et al. (2019) [122]	Preeclampsia between 34–37 weeks	RCT	England and Wales. Multi-site including 46 maternity units	N = 901 To compare planned birth (usually IOL) (n = 448) versus EM (n = 338) in women with late preterm pre-eclampsia from 34 to 37 weeks and a singleton or dichorionic diamniotic twin pregnancy.	Planned birth reduced maternal morbidity and severe hypertension (65% vs 75%, RR = 0.86, 95% CI 0·79–0·94; p = 0·0005), but more neonatal admissions for prematurity (42% vs 34%, RR 1·26, 1·08–1·47; p = 0·0034)	Low
Churchill et al. (2013)^†^ [119]	Hypertension—Severe preeclampsia between 24 and 34 weeks	Cochrane review of RCTs; quasi randomised studies were excluded	4 included RCTs from Europe, USA and South Africa	N = 425 To compere planned early birth (by IOL or CS) (n = 222) versus EM (n = 203)	An expectant approach may be associated with decreased morbidity for the baby. There was insufficient data for reliable conclusions about the comparative effects on most outcomes for the mother.	Low
Vigil-De-Gracia et al. (2013) [121]	Hypertension—Severe preeclampsia between 28 and 33 weeks	RCT	Latin America. Multisite study including 8 tertiary teaching hospitals between 2010 and 2012	N = 267 To compare planned early birth (n = 133) versus EM (n = 134)	EM was not associated with neonatal benefit and may increase the risk of abruption and small for gestational age.	Low
Cluver et al. (2017) [118]	Hypertension—all forms from 34 weeks to term	Cochrane review of RCTs; studies with quasi randomised design or cross-over design were excluded	5 included RCTs from the Netherlands, India; USA, Saudi Arabia and Egypt (including Hypitat I and II)	N = 1819 To compare planned early birth (n = 915) versus EM (n = 904)	Planned early birth is associated with less composite maternal morbidity and mortality. There is no clear difference in the composite outcome of infant mortality and severe morbidity; however, this is based on limited data (from two trials) assessing all hypertensive disorders as one group.	Low
Tajik et al. (2012) [211]	Hypertension and mild preeclampsia between 36 and 41 weeks	Post hoc analysis of RCT (HYPITAT-I)	The Netherlands.	N = 756 (IOL group = 377; EM group = 379) To assess whether cervical ripeness should play a role in the decision for IOL.	The superiority of IOL in preventing high-risk situations varied significantly according to cervical favourability.	Low
Walker et al. (2016) [171]	Maternal age	RCT	UK. Multi-centre study including 39 Centers between August 2012 to March 2015	N = 619 To test if IOL at 39 weeks reduces CS rates for nulliparous women of advanced maternal age (≥ 35) by comparing outcomes for IOL (n = 305) with EM (n = 314). Women who had undergone in vitro fertilization with the use of donor eggs were excluded.	No significant differences in the two groups in terms of CS rates (32% in IOL group vs 33% in EM group; RR 0.99), % of women who had a vaginal birth with the use of forceps or vacuum (38% vs 33%, RR 1.30), the women’s experience of childbirth, or adverse maternal or neonatal outcomes. There were no maternal or infant deaths.	Low
Boulvain et al. (2016)^‡^ [174]	Macrosomia—Suspected	Cochrane review of RCTs between 1995 and 2015; studies with quasi randomised design or cross-over design were excluded	4 included RCTs that include participants from France, Switzerland Belgium, Israel, USA and UK.	N = 1190 To compare outcomes associated with IOL (n = 590) versus EM (n = 600) for suspected fetal macrosomia between 37 to 40 weeks in non-diabetic women.	IOL had no clear effect on the risk of CS (RR 0.91) or instrumental birth (RR 0.86), but did reduce shoulder dystocia (RR 0.60) and fracture (any) (RR 0.20). There was no strong evidence of any difference between groups for measures of neonatal asphyxia: low infant Apgar scores (<7 at 5 minutes) (RR 1.51) or low arterial cord blood pH (RR 1.01). There was no perinatal mortality, and no differences in the groups in terms of portion of newborns with intraventricular haemorrhage (RR 1.06), nor neonatal intensive care admissions (RR 0.66).	Low
Keulen et al. (2018) [34]	Post-term pregnancy	Systematic review	Reviewed evidence from RCTs included in Cochrane review by Middleton, Shepherd [6]	N = 4 RCTs To compare outcomes associated with IOL at 41 weeks versus 42 weeks.	The incidence of potentially gestational age associated perinatal mortality between 41 and 42 weeks was 0/2.444 (0%) for the IOL group versus 4/2.452 (0.16%) in the EM group (number needed to treat was 613). This review concluded that there is not sufficient evidence for IOL at 41 weeks instead of 42 weeks.	Low
Keulen et al. (2019) [35]	Post-term pregnancy	RCT	The Netherlands. Multi-site study including 123 primary care midwifery practices and 45 hospitals with data collected between 2012 and 2016.	N = 1801 low risk women with an uncomplicated singleton pregnancy. To compare IOL at 41 weeks (n = 900) versus EM until 42 weeks (n = 901)	IOL was associated with reduced adverse perinatal outcomes (1.7% vs 3.1%, absolute risk difference of 1.4%, 95% CI -2.9 to 0.0, p = 0.22 for non-inferiority). No significant difference was found in composite adverse maternal outcomes or CS rates.	Low
Middleton et al. (2018) [6]	Post-term pregnancy	Cochrane review. Cluster RCTs, quasi-RCTs, or cross-over design were excluded.	30 RCTs (1969–2015) from Norway, China, Thailand, the USA, Austria, Turkey, Canada, UK, India, Tunisia, France, Finland, Spain, Sweden and the Netherlands.	N = 12,479 To assess the effects of a policy of IOL at or beyond term compared with a policy of awaiting spontaneous labour (or until planned birth becomes required) on pregnancy outcomes for infant and mother. Only trials including women at low risk for complications were included.	IOL was associated with fewer perinatal deaths (RR) 0.33) (2 vs 16), lower NICU admissions (RR 0.88), fewer babies had Apgar scores <7 at five minutes (RR 0.70), and fewer CS (RR 0.92). The number needed to treat to in order to prevent one perinatal death was 426. There was no significant difference between groups for perineal trauma (RR 1.09), postpartum haemorrhage (RR 1.09), length of maternal hospital stay, or neonatal trauma (RR 1.18). IOL was associated with an increase in operative vaginal births (RR 1.07), in particular for IOL at < 41 weeks.	Low
Bond et al. (2017) [82]	PROM, preterm	Cochrane review of RCTs; quasi RCTs were excluded	USA, the Netherlands, Mexico, Albania, Australia, New Zealand, Argentina, South Africa, Brazil, UK, Norway, Egypt, Uruguay, Poland, and Romania	N = 3617 To compare outcomes associated with planned early birth by CS or IOL with EM for women with PROM of<37 weeks with no maternal or fetal contraindications to EM.	In terms of neonatal outcomes, there were no clear differences in neonatal sepsis (RR 0.93), proven neonatal infection with positive blood culture (RR 1.24), and overall perinatal mortality (RR 1.76), but found that early birth was associated with a higher rate of neonatal death (RR 2.55), respiratory distress syndrome (RR 1.26), need for ventilation (RR 1.27), and NICU admission (RR 1.16). In terms of maternal outcomes, early birth was associated with an increased rate of CS (RR 1.26) and increased rate of endometritis (RR 1.61), and reduced incidence of chorioamnionitis (RR 0.50).	Low
Middleton et al. (2017) [81]	PROM, at term	A Cochrane review of randomised and quasi-randomised controlled trials	23 RCTs (1990–2015) from Pakistan, China, Scotland, Canada, the UK, Australia, Israel, Sweden and Denmark, Brazil, Canada, Denmark, Germany, India, Norway, Serbia, Sweden, the Netherlands, Turkey, USA, and Zimbabwe.	N = 8615 To compare IOL (immediate or within 24 hours) with EM (no planned intervention within 24 hours) for women with PROM of ≥37 weeks with no maternal or fetal contraindications to EM.	Women who had IOL were at a reduced risk of maternal infectious morbidity (chorioamnionitis and/or endometritis) than women who had EM (RR 0.49), and their neonates were less likely to have early-onset neonatal sepsis (RR 0.73). No clear differences were seen for the risk of CS (RR 0.84); serious maternal morbidity or mortality (no events); definite early-onset neonatal sepsis (RR 0.57); or perinatal mortality (RR 0.47).	Low
Bond et al. (2015) [185]	Suspected fetal compromise, incl. intrauterine growth restriction and oligohydramnios	A Cochrane review of randomised and quasi randomised controlled trials	3 RCTs with participants from the Netherlands and Sweden	N = 546 To assess the effects of early birth (n = 269) versus EM (n = 277) of suspected compromised fetus at term (≥ 37 weeks) on neonatal, maternal and long-term outcomes.	There are no major differences in major neonatal and maternal outcomes between the two groups.	Low
Stock et al. (2016) [186]	Suspected fetal compromise	A Cochrane review of RCTs; studies with quasi randomised design or cross-over design were eligible but non identified	1 RCT included conducted between 1993–2001 in 69 hospitals in 13 countries (Belgium, Cyprus, Czech Republic, Germany, Greece, Hungary, Italy, Netherlands, Poland, Portugal, Saudi Arabia, Slovenia, UK).	N = 548 women (588 babies) To assess the effects of immediate (n = 296) versus deferred (n = 291) birth of preterm babies (24–36 weeks) with suspected fetal compromise (and uncertainty about whether to deliver early or not) on neonatal, maternal and long-term outcomes.	For preterm babies with suspected compromise and uncertainty about whether to deliver or not, there appears to be no benefit to immediate birth.	Low
Dodd et al. (2014) [156]	Twin pregnancy	Cochrane review of RCTs; studies with cross-over design were excluded	2 included RCTs. A multi-site study across Australia, New Zealand and Italy, and a single site study from Japan.	N = 271 and 542 infants To compare elective birth by CS or IOL from 37 week (n = 133) with EM (n = 138) for women with an otherwise uncomplicated twin pregnancy. EM = IOL after 38 weeks, spontaneous onset of labour, or CS close to 39 weeks.	No statistically significant differences in CS, perinatal death or serious infant morbidity, or maternal death or serious maternal morbidity.	Low

^†^review by Wang et al. (2017) [143] included the same studies.

^‡^review by Magro-Malosso (2017) [177] included the same studies with similar findings.

EM = expectant management; GDM = Gestational diabetes mellitus; IOL = Induction of labour; CS = Caesarean section; NICU = neonatal intensive care unit

**Table 2:** Included non-randomised studies.

Author, Publication year	Indication	Study design	Country and Setting	Aim and Participants	Main Findings	Risk of bias
Kohari et al. (2017) [165]	Cholestasis	Retrospective cohort study	USA. Single centre study with data from between 2005 and 2013	N = 186 To compare outcomes for women who birthed under an active management policy (2009–2013) versus those who birthed prior to the introduction of this policy (2005–2008). All women with bile acids >40 μmol/L and diagnosis <36 weeks were included.	The active management policy was found to be associated with a significant reduction in the incidence of stillbirth (0% versus 3.4%, p = 0.035). There was no difference in the age at birth, CS rates or NICU admissions.	Moderate
Puljic et al. (2015) [164]	Cholestasis	Retrospective cohort study	USA. Analysis of a national dataset of 1,604,386 women between 34 and 40 weeks in California between 2005 and 2008.	N = 5545 (identified with cholestasis). To characterise the risk of infant and fetal death by each additional week of EM versus immediate birth in pregnancies complicated by cholestasis.	Birth at 36 weeks gestation was associated with lower perinatal mortality.	Low
Bettikher et al. (2016) [147]	Diabetes—GDM	Retrospective cohort study	Russia. Single centre study with data from 2014.	N = 231 To evaluate the outcomes of IOL (n = 43) versus spontaneous labour (n = 188) in women with GDM. No details provided on the baseline characteristics of the two groups.	No significant difference between the two groups in terms of CS rate, the frequency of uterine inertia, uncoordinated contractions or foetal distress.	Serious
Feghali et al. (2016) [148]	Diabetes—GDM	Retrospective cohort study	USA. Single centre study with data from January 2009-October 2012	N = 863 To compare CD rates in women undergoing IOL at each week of gestation with EM to a later gestational age. Women with previous CS or other complications were excluded.		Moderate
Grabowska et al. (2017) [149]	Diabetes—GDM	Retrospective cohort study	Poland. Single centre study with data from 2013–2014	N = 204 To compare the mode birth for women with GDM who underwent IOL (n = 96) versus those with spontaneous onset of labour (n = 32).	IOL did not increase the risk for CS (25% versus 25%, p = 0.66).	Moderate
Hochberg et al. (2019) [152]	Diabetes—GDM	Retrospective cohort study	Israel. Single site study with data from between 2014 and 2016	N = 430 To compare maternal and neonatal outcomes in women with good glycemic controlled gestational diabetes mellitus (GDM) undergoing IOL at 37 + 0–38 + 6 weeks (n = 193) versus 39 + 0–40 + 6 weeks (n = 237)	Rates of composite maternal outcome and composite neonatal outcome did not differ between groups. There were higher rates of hypertensive complications of any kind and pre-eclampsia, in women induced at early term (11.04% vs. 4.26%, *p* = 0.021, and 5.92% vs. 1.60%, *p* = 0.04, respectively).	Moderate
Melamed et al. (2016) [150]	Diabetes—GDM	Retrospective cohort study	Canada. National dataset of all birth between April 2012 and March 2014	N = 6417 To compare outcomes for those who underwent IOL at 38 or 39 weeks (for reasons related to GDM) with those who were managed expectantly. Women with comorbid conditions or a previous CS were excluded.	IOL at 38 or 39 weeks was associated with a lower CS rates but higher risk of NICU admission when done at <39 weeks of gestation	Moderate
Vitner et al. (2019) [151]	Diabetes—GDM	Retrospective cohort study	Israel. Single site study with data analysed from between 2007–2014	N = 880 To compare IOL at each week of gestation versus EM for GDM	IOL was associated with increased risk for adverse composite neonatal outcome or NICU admission when done prior to 39 weeks. IOL at 37 weeks was associated with adverse composite neonatal outcome (aOR 2.2, 95% CI 1.4–3.6) and NICU admission (aOR 2.5, 95% CI 1.4–4.4). At 38 weeks, with NICU admission (aOR 2.0, 95% CI 1.4–2.9), and at 39 weeks with fracture of the clavicle.	Moderate
Al-Kaff et al. (2015) [183]	Gastrochisis	Retrospective cohort study	Canada. Analysis of Canadian Paediatric Surgery Network national database from 2005–2013.	N = 519 infants To assess the effect of mode and timing of birth for fetal gastroschisis on neonatal outcomes by comparing outcomes for mode of birth (spontaneous labour, n = 190; IOL, n = 280; CS, n = 49) and timing of birth (≤35 weeks, n = 8; 36–37 weeks, n = 193; ≥38 weeks, n = 69).	Neither mode nor timing of birth were associated with significant benefits or adverse effects.	Low
Alanis et al. (2008) [127]	Hypertension—Severe preeclampsia between 24 and 34 weeks of gestation	Retrospective cohort study	USA. Single centre study with data from between 1996 and 2006	N = 491 To describe the success rate of and analyse differences in neonatal outcomes with IOL (n = 282) versus planned CS (n = 209)	Neonatal outcomes were not worsened by IOL although it was rarely successful at under 28 weeks of gestation.	Moderate
*Alexander et al. (1999) [130]	Hypertension—Severe preeclampsia -very low birth weight infants (weights between 750 and 1500g)	Retrospective cohort study	USA. Single centre study with data from 1988 to 1997	N = 278 To compare IOL (n = 145) with planned CS (n = 133)	IOL in cases of severe pre- eclampsia is not harmful to very low birth weight infants.	Moderate
Amorim et al. (2015) [124]	Hypertension—Severe preeclampsia	Prospective cohort study	Brazil. Single centre study between August 2008 and July 2009	N = 500 To compare vaginal birth (n = 159) and CS (n = 341) in terms of maternal outcomes. Labour was spontaneous in 110 patients (22%) and induced in 141 (28.2%)	CS was associated with severe maternal morbidity, irrespective of the presence of labour. The authors suggest that Induction of labour should be considered a feasible option in these patients.	Moderate
*Blackwell et al. (2001) [131]	Hypertension—Severe preeclampsia at < = 34 weeks	Case control study	USA. Single centre study with data from January 1991 to December 1998	N = 215 To examine the success rate of IOL, identify factors associated with its success and evaluate neonatal outcomes based on induction success or failure. To compare outcomes associated with planned CS (n = 64), CS following attempted IOL (n = 82), and vaginal birth following successful IOL (n = 69).	Induction success was significantly associated with gestational age. While, attempted IOL did not appear to increase neonatal morbidity, induction was rarely successful at <28 weeks.	Moderate
Ertekin. Et al. (2015) [125]	Hypertension—Severe preeclampsia between 27 and 34 weeks	Prospective cohort study	Turkey. Single centre study from 2010 to 2012	N = 70 To compare EM (n = 33) versus early birth (n = 37) on the first year of neurologic development of infants, and other neonatal and maternal outcomes	There was no significant difference in the first year neurological development of infants between the two groups. The women’s average weeks of gestation were 31.09 ± 2.53 in the EM group and 30.64 ± 2.31 in the immediate birth group.	Moderate
*Mashiloane et al. (2002) [126]	Hypertension—Severe preeclampsia from 26 to 32 weeks	Prospective cohort Study	South Africa. Single-centre data from June 1999 to June 2000	N = 108 To compare outcomes associated with planned CS (n = 68), CS following attempted IOL (n = 14), and vaginal birth following successful IOL (n = 26).	Perinatal mortality was highest for the babies delivered following IOL (vaginal birth vs. CS after IOL, P = 0·0004; vaginal birth vs. planned CS, P = 0·002).	Moderate
*Nassar et al. (1998) [132]	Hypertension—Severe preeclampsia between 24 and 34 weeks	Case control study	USA. Single-centre data from 1^st^ January 1992 to 31 December 1996	N = 306 To determine the rate of vaginal birth after IOL in severe preeclampsia remote from term and to discover potential predictors of successful IOL. To compare outcomes associated with planned CS (n = 161), CS following attempted IOL (n = 75), and vaginal birth following successful IOL (n = 70).	48% of patients given the chance successfully delivered vaginally. The median Bishop score was significantly higher (3 vs 2, P = .004) and the total hospital stay was significantly shorter in the vaginal birth after IOL than in the CS after IOL. There were no significant differences between the 2 groups in gestational age at birth, birth weight, 5-minute Apgar score, or postpartum endometritis. Only the Bishop score was significantly associated with a successful IOL (OR 1.38). Gestational age reached marginal significance (OR 1.30).	Moderate
Cruz et al. (2012) [129]	Hypertension—gestational	Retrospective population based cohort study	USA. Data from multicentre database (from 12 clinical centres and 19 hospitals) with 228,668 deliveries greater than 23 weeks, from between 2002 and 2008, with the majority (87%) of births occurring from 2005 through 2007	N = 3588 Assess the optimal timing of birth for women with gestational hypertension by quantifying the risks of adverse maternal and fetal outcomes associated with IOL at each gestational week, from 36 to 41 completed weeks, compared with those with ongoing pregnancy.	IOL between 38- and 39-weeks’ balances the lowest maternal and neonatal morbidity/mortality. After IOL, the rate of maternal morbidity/mortality reached a nadir of 89.9 per 1000 live births (95% confidence interval, 68.1–111.8) at 38–38 6/7 weeks’ gestation, although the rate of neonatal morbidity/mortality fell to 10.5 per 1000 live births (95% confidence interval, 2.8–18.2) at 39–39 6/7 weeks. There were only 3 total stillbirths in the study cohort.	Moderate
Hutcheon et al. (2010) [128]	Hypertension—pre-existing (gestation is variable)	Retrospective population based cohort study	USA. Data from the US National Centre for Health Statistics’ period-linked birth infant death and stillbirth files from between 1995 and 2005	N = 171 669 singleton births to women with pre-existing (chronic) hypertension. To determine the optimal timing of birth by quantifying the gestational age-specific risks of stillbirth associated with ongoing pregnancy and the gestational age-specific risks of neonatal mortality or serious neonatal morbidity following the IOL.	IOL between 38- and 39-weeks’ balances the lowest maternal and neonatal morbidity/mortality. The risk of stillbirth remained stable at 1.0–1.1 per 1000 ongoing pregnancies until 38 weeks, before rising steadily to 3.5 per 1000 at 41 weeks. The risk of serious neonatal morbidity/neonatal mortality decreased sharply between 36 and 38 weeks from 137 to 26 per 1000 induced births, before stabilising beyond 39 weeks.	Serious
Knight et al. (2017) [172]	Maternal age	Retrospective cohort study	UK. Multi-site study with national public hospital data from between April 2009 and March 2014	N = 77327 To compare outcomes between IOL (at between 39 and 41 weeks) (n = 51,744) and EM (n = 25,583) for nulliparous women aged ≥ 35. Women with pre-existing comorbidities or other indications for induction were excluded.	IOL at 40 weeks was associated with a lower risk of in-hospital perinatal death (0.08% vs 0.26%; RR 0.33; p = 0.015) and meconium aspiration syndrome (0.44% vs 0.86%; RR 0.52; p = 0.002), but an increased risk of instrumental vaginal birth (RR 1.06; p = 0.020) and CS (RR 1.05; p = 0.019). A number needed to treat analysis indicated that 562 IOL at 40 weeks would be required to prevent one perinatal death.	Low
Oron, Hirsch [173]* [173]	Maternal cardiac disease	Prospective cohort study	Israel. Single centre high risk pregnancy clinic data from 1995 to 2001.	N = 121 To examine the safety of IOL in women with heart disease by comparing outcomes for women who underwent IOL between 37 and 40 weeks (n = 47) versus EM (n = 74)	There was no significant difference in complication rate between the two groups.	Moderate
Kawakita et al. (2017) [167]	Maternal Elevated BMI	Retrospective cohort study	USA. Secondary analysis of data from the Consortium on Safe Labor, conducted from 2002 to 2008 in 12 clinical centers.	N = 4349 (morbidly obese women) To compare the CS rate of elective IOL with EM in morbidly obese women (≥40 kg/m2) with singleton, cephalic gestations and no previous CS or other comorbidity between 37 and 41+6 weeks.	In nulliparas, elective IOL was not associated with increased risks of CS and was associated with decreased risks of macrosomia (2.2% vs 11.0%) at early term (37 0/7 to 38 6/7) and decreased NICU admission (5.1% vs 8.9%) at full term (39 0/7 to 40 6/7). In multiparas, IOL was associated with a decreased risk of macrosomia at early term (4.2% vs 14.3%), CS at full term (5.4% vs 7.9%), and composite neonatal outcome (0% vs 0.6%) at full term.	Low
Lee et al. (2016) [169]	Maternal Elevated BMI	Retrospective cohort study	USA. All birth in California in 2007 using a national dataset.	N = 74,725 (obese women) To compare outcomes between elective IOL and EM in obese women (≥30.0 kg/m2) with singleton pregnancies between 37–40 weeks. Women with prior CS or chronic diseases were excluded.	IOL was associated with lower CS rates, and lower odds of macrosomia. There were no differences in the other reported outcomes.	Low
Pickens et al. (2018) [168]	Maternal Elevated BMI	Retrospective cohort study	USA. All birth in California between 2007 and 2011 using a national dataset.	N = 165,975 (obese women) To compare outcomes associated with IOL versus EM in obese women (≥30.0 kg/m2) with singleton pregnancies between 39 and 41 weeks. Women with prior CS or medical comorbidities were excluded.	IOL was associated with reduced CS rates (at 39 weeks of gestation, frequencies were 35.9% vs 41.0%, p = < .05), reduced severe maternal morbidity (5.6% vs 7.6%, p = < .05), and reduced NICU admissions (7.9% vs 10.1%, p = < .05).	Low
Wolfe et al. (2014) [170]	Maternal Elevated BMI	Retrospective cohort study	USA. Single centre study with data from between 2007 and 2012	N = 470 To compare outcomes in obese (≥30.0 kg/m2) nulliparous women with an unfavourable cervix (modified Bishop score < 5) undergoing elective IOL between 39 and 41 weeks (n = 60) with EM after 39 weeks (n = 410). Women with medical comorbidities were excluded.	IOL was associated with higher rates of CS (40% vs 25.9%, p = .022), and NICO admission rates (18.3% vs 6.3%, p = .001). Other maternal and neonatal outcomes were similar.	Low
Cheng et al. (2012) [176]	Macrosomia	Retrospective cohort study	USA. Analysis of national dataset—Vital Statistics Natality birth certificate registry provided by the Centre of Disease Control and Prevention over a one year period (2003).	N = 132,112 To compare the frequency of CS for women who had an IOL at 39 weeks with a neonatal birthweight of 4000 +/- 125g (birthweight 3875-4125g) with women who gave birth (following IOL or spontaneous onset of labour) at 40 weeks with birthweight 4075–4325 g, at 41 weeks with a birthweight at 4275–4525 g, or 42 weeks with a birthweight of 4475–4725 g	The frequency of CS in the IOL group was lower compared with women who delivered at a later gestational age (35.2% versus 40.9%; OR 1.25)	Low
Sanchez-Ramos et al. (2002)* [175]	Macrosomia—Suspected	Systematic review and meta-analysis. Only observational studies reported here as RCTs included in Cochrane review.	11 studies included including 2 RCTs and 9 observational studies from the US (5), Denmark (2) and Germany, Israel, Italy, and Norway (1). Studies were published between 1966 and 2002.	N = 3751 To compare outcomes associated with IOL (n = 2700) versus EM (n = 1051) for suspected macrosomia	Analysis of non-randomised studies indicates that the risk of CS may be increased when IOL is undertaken. Women who experienced spontaneous onset of labour had a lower incidence of CS (OR 0.39) and higher rates of spontaneous vaginal birth (OR 2.07). No differences were found in rates of operative vaginal deliveries, incidence of shoulder dystocia, or abnormal Apgar scores in the analyses of the observational studies.	Low
Bleicher et al. (2017) [41]	Post-term pregnancy	Retrospective cohort study	Israel. Single centre study comparing data between two policy periods	N = 1930 To compare outcomes for women who birthed under a policy of IOL at 42 weeks (n = 968; from2008–2009) with those who birthed under a policy of IOL at 41 weeks (n = 962; from 2012–2013).	Both the overall CS rate as well as the CS rate for women who underwent IOL was lower during the 41-policy period than during the 42-policy period (15% vs 19.4%, p = 0.0135 and 19% versus 27%, p = 0.0067). IOL at 41 weeks was also associated with a significant reduction in 1st and 2nd degree perineal lacerations and neonatal readmission within 30 days of discharge.	Moderate
Daskalakis et al. (2014) [40]	Post-term pregnancy	Retrospective cohort study	Greece. Single centre study with data from between September 2009 and September 2011	N = 483 To compare outcomes associated with IOL at 41+1 (n = 211) versus EM until spontaneous onset of labour or IOL at 42 weeks (n = 227). Women with previous CS and comorbidities were excluded.	No significant differences in the two groups in terms of CS rate (36.5% vs 34.4%) or operative vaginal birth (11.4% vs 9.2%).	Moderate
Haq et al. (2012) [36]	Post-term pregnancy	Prospective cohort study	Pakistan. Single centre study with data from between 2006 and 2008.	N = 78 To compare CS rates for IOL at 40 weeks (n = 39) versus 41 weeks (n = 39).	Less women induced at 41 weeks had a CS compared to the 40 weeks group (16% vs 29%, p < 0.0001).	Moderate
Hermus et al. (2009) [39]	Post-term pregnancy	Retrospective matched cohort study	Netherlands. Multi-centre study including two hospitals, with data from between 2002 and 2005.	N = 674 To compare outcomes for women who underwent IOL (= 377) at 42 weeks to EM beyond 42 week (n = 377). Women with comorbidities were excluded.	EM was associated with lower rate of CS (12.5% vs 13.6%, RR 0.9), but higher incidence of shoulder dystocia (RR, 4.3) and meconium-stained amniotic fluid (RR, 1.8).	Moderate
Kassab et al. (2011) [38]	Post-term pregnancy	Retrospective cohort study	UK. Single centre study comparing data between two policy periods	N = 351 To compare outcomes associated with a policy period of IOL at 41+3 days (n = 124; August 2006 and March 2007) versus a policy-period of IOL at 42 weeks (n = 227; April 2007 and July 2008). Women with previous CS or comorbidities were excluded.	The CS rate was higher under the earlier IOL policy (p = 0.04) for nulliparous women only. The average delay in birth was >2 days. The study was not powered to examine neonatal outcomes.	Moderate
Mahomed et al. (2016) [37]	Post-term pregnancy	Retrospective cohort study	Australia. Multisite data from Queensland Perinatal Data Collection Unit. All births in Qld that meet inclusion criteria between January 2005 and December 2012	N = 7,811 To compare CS rates associated with IOL at 40–40+6 weeks (n = 2153) versus spontaneous birth at 41–41+6 weeks (n = 5658)	CS rates were significantly higher in the IOL group (OR 1.52; 21% versus 14.9%).	Moderate
McCoy et al. (2018) [47]	Post-term pregnancy	Secondary analysis of prospective cohort study	USA. Single centre study with data from between May 2013 to June 2015	N = 854 To compare outcomes associated with IOL at term (37–40+6) (n = 700) versus IOL at >41 weeks (n = 154) among women with an unfavourable cervix (Bishop score of <6 and cervical dilation <2 cm). Women who had had a previous CS or contraindication to vaginal birth excluded.	Women induced at >41 weeks had an increased risk of CS versus those induced at term (46.8 versus 26.0%, p < .001).	Moderate
Mya et al. (2017) [46]	Post-term pregnancy	Secondary analysis two WHO multi-country surveys between 2004–2008	A secondary analysis of the WHO Global Survey (WHOGS) and the WHO Multi-country Survey (WHOMCS) conducted in Africa, Asia, Latin America and the Middle East, from 292 facilities in 21 countries.	N = 31,052 To assess outcomes of IOL (n = 4,332) in comparison to EM (n = 26,720) at and beyond 41 weeks. Only women with low risk singleton pregnancies at ≥41 completed weeks were included.	Compared to IOL, EM was significantly associated with decreased risk of CS in both databases (OR 0.70 and IOR 0.67). The choice between IOL and EM should be cautiously considered since the available evidences are still quite limited.	Low
Pavicic et al. (2009) [42]	Post-term pregnancy	Retrospective cohort study	Canada. Single centre study with data from 2005 to 2007	N = 1367 To compare outcomes associated with IOL at 41+1 (n = 722) versus EM until spontaneous onset of labour or IOL at 42 weeks (n = 645). Exclusion criteria not mentioned.	IOL was significantly associated with increased risk of CS (25.4% vs 16.6%, p = 0.001).	Serious
Teo and Kumar (2017) [43]	Post-term pregnancy	Retrospective cohort study	Australia. Single centre study with data from 2007 to 2013	N = 6501 To compare outcomes associated with IOL at 41+1 (n = 3588) versus EM (n = 2913). Women with commodities were not excluded, and women in the IOL were more likely to be obese or hypertensive.	IOL was associated with higher rates of CS (29.4% vs 18.5%, p = 0.001) and instrumental birth (20.2% vs 17.7%, p = 0.012)	Serious
Thangarajah et al. (2016) [44]	Post-term pregnancy	Retrospective cohort study	Germany. Singe centre study with data from between 2000–2014	N = 856 To compare outcomes associated with IOL at 41+1 (n = 400) versus EM (n = 456). Women with previous CS or comorbidities were excluded.	IOL was associated with increased rates of CS (33.8% vs. 21.1%, p < 0.001), and perineal lacerations (38.1% vs 26.4%, p = 0.002).	Moderate
Wolff et al. (2016) [45]	Post-term pregnancy	Retrospective cohort study	Denmark. Multi-site study including 24 centres between 2009–2012.	N = 36,837 To compare outcomes for women who birthed under a policy of IOL at 41+2 weeks (2012) (n = 8545) versus those who birthed prior to the introduction of this policy and birthed under a policy of IOL at 42 weeks (2010) (n = 9713). Women with comorbidities were excluded.	The number of IOL within the study population doubled after implementation of the new guideline. There was a significant reduction in CS rates between 2010 and 2012 (p = 0.05), and a non-significant reduction in perinatal mortality of 60% (from 10 to 3). There were no significant differences in instrumental deliveries or perinatal outcomes.	Moderate
Omole-Ohonsi et al. (2009) [83]	PROM, at term	Prospective cohort study	Nigeria. Single centre data with data collection commenced in 2004 (end date not stated)	N = 200 To compare immediate IOL (N = 100) with delayed IOL after EM for 12 hours (N = 100) for women with PROM ≥ 37 weeks and no contraindication to IOL or vaginal birth.	33% of the women in the delayed IOL group went into spontaneous labour. Immediate IOL was associate with significantly lower rates of CS (OR = 0.18), and operative vaginal birth (OR = 0.26), and higher rates of vaginal birth (OR = 6.10). There was no significant difference in the neonatal outcomes.	Low
Pintucci et al. (2014) [85]	PROM, at term	Retrospective cohort study	Italy. Single centre data from between January 2006 and December 2008	N = 1315 To analyse outcomes associated with a policy of delayed IOL after EM for 48 hours for women with PROM ≥ 37 weeks and no other obstetric risk factors.	84% of the women went into spontaneous labour within 48 hours. There were very low rate of clinical chorioamnionitis (2.3%) and neonatal infection rate (2.8%). The overall CS rate was 4.5%, which was lower for women who went into labour spontaneously than those who underwent IOL (OR = 1.76).	Moderate
Sadeh-Mestechkin et al. (2016) [84]	PROM, at term	Retrospective cohort study	Setting not specified. Single centre data from March 2013 to April 2014	N = 325 To compared immediate IOL (N = 213) versus delayed IOL after EM for 48 hours (N = 112) for women with PROM ≥ 37 weeks	The delayed group had significantly higher rate of prolonged hospitalisation (p = 0.043), and higher rates of CS (16.4% vs 7.1%, p = 0.024). There was no significant difference in chorioamnionitis, postpartum endometritis, and there were no cases of early neonatal sepsis.	Moderate
Brzezinski-Sinai et al. (2018) [187]	Suspected fetal compromise, oligohydramnios	Retrospective cohort study	Israel. Single centre study with data between 1991–2011	N = 144 To compare outcomes for women with isolated oligohydramnios between 34 and 36.6 weeks who laboured spontenaousely (n = 33) versus those who underwent IOL (n = 111). Included all singleton pregnancies diagnosed with isolated oligohydramnios following a definition of amniotic fluid index [AFI]<5 cm. Excluded pregnancies with other complications.	Spontaneous labour was associated with statistically significant higher rates of CS (p < .001), as well as higher rates of maternal infection, chorioamnionitis, and transitory tachypnoea of the newborn. The study concludes that IOL may be beneficial to both the neonate and the mother.	Moderate
Rabinovich et al. (2018) [188]	Suspected fetal compromise, intrauterine growth restriction	Retrospective cohort study	Israel. Single centre study.	N = 2232 To compare outcomes for IOL (n = 1428) versus EM (n = 804) for IUGR between 34 and 38 weeks	IOL was associated with lower stillbirth and neonatal death rates (p < .001), higher 1 and 5 min Apgar scores and a higher vaginal birth rate. IOL at 37 weeks protected from stillbirth but not from adverse composite neonatal outcomes.	Moderate
de Castro et al. (2016) [158]	Twin pregnancy	Retrospective cohort study	Israel. Single centre study with data from between 2004 and 2011	N = 883 To determine the success rate of a trial of labour in twin pregnancies, and identify factors that may affect the chances of success by comparing outcomes for IOL (n = 287; 188 (non-Foley) + 99 (Foley) with spontaneous vaginal birth for both twins (N = 530), a CS (N = 51) or a vaginal birth for first twin and CS for 2^nd^ twin (N = 15). All twin pregnancies, first twin cephalic who had not had a previous CS were included.	IOL significantly decreased the chance for achieving vaginal birth (Foley catheter induction 74.7%; non-Foley induction 86.7%; no induction 88.9%, P < 0.001). No significant difference in terms of 5-minute Apgar score in trial of labour versus CS group.	Serious
Hamou et al. (2016) [159]	Twin pregnancy	Retrospective cohort study	Israel. Single centre hospital data over a 20 year period	N = 4605 To determine the efficacy of IOL for twin pregnancy by comparing outcomes for women who had an IOL (n = 653); spontaneous birth (n = 2937) versus elective CS (n = 1015). All twin gestation who delivered after 24 complete gestation weeks were included. 25% of spontaneous twin births occurred in early preterm, < 34 weeks.	IOL was associated with a lower rate of CS than those who come with spontaneous labour (77% reduction, OR 0.23). This study also found that the IOL was associated with lower rates of neonatal death (78% reduction, OR 0.22). The rate of vaginal birth in the IOL group was 81%.	Serious
Jonsson (2015) [157]	Twin pregnancy	Retrospective cohort study	Sweden. Medical records from two university hospitals from 2004–2013 in Uppsala and 1994–2013 in Örebro.	N = 462 To investigate the association between IOL and CS in twin pregnancies ≥34 weeks by comparing CS rates for women who received IOL (n = 220) with those who had spontaneous onset of labour (n = 242). Women with a history of previous CS were excluded.	IOL increases the risk of CS compared with spontaneous labour onset (21% versus 12%), especially if Foley catheter or prostaglandins are required. However, approximately 80% of induced labours were delivered vaginally. There were no differences in Apgar scores	Low
Tavares et al. (2017) [160]	Twin pregnancy	Retrospective cohort study	Portugal. Data from single centre database with 288 twin pregnancies between January 2007 and December 2011.	N = 75 To compare outcomes for IOL (n = 33) versus spontaneous vaginal birth (n = 44) in uncomplicated twin pregnancy after 36 weeks of gestation.	This study found no statistical differences between the two groups in terms of maternal and neonatal morbidity, and admission to the NICU. There was an increased incidence of CS after IOL (60.6 vs. 33.3%, p < .05).	Low

*included in review by Mozurkewich, Chilimigras (10).

EM = expectant management; IOL = Induction of labour; CS = Caesarean section; GDM = Gestational diabetes mellitus; NICU = neonatal intensive care unit

Included studies are listed in Tables 1 and 2. We did not identify any studies for inclusion in relation to fetal alloimmune disease or Rh disease, antepartum haemorrhage, (suspected) chorioamnionitis, or fetal death and only one study in relation to ‘other’ maternal medical indications, about maternal cardiac disease. The evidence for each indication for IOL is summarised below.

### Post-term pregnancy

There were 15 studies related to IOL for post-term pregnancy (> 40 weeks). These included a Cochrane review [6], a systematic review [34], an RCT [35], a prospective cohort study [36], nine retrospective cohort studies [37–45], and two secondary analyses of cohort studies [46, 47]. The Cochrane review assessed the effects of a policy of IOL at or beyond term compared with a policy of awaiting spontaneous labour (or until planned birth is deemed necessary) on maternal and neonatal outcomes [6]. This review includes 30 RCTs, with 12,479 women [48–77]. The majority of trials (about 75% of participants) adopted a policy of IOL at ≥ 41 weeks. IOL was associated with fewer perinatal deaths (2 vs 16) (risk ratio (RR) 0.33, 95% confidence interval (Cl) 0.14 to 0.78), lower NICU admissions (RR 0.88, 95% Cl 0.77 to 1.01), fewer babies with Apgar scores <7 at five minutes (RR 0.70, 95% Cl 0.50 to 0.98), and fewer CS (RR 0.92, 0.92, 95% Cl 0.85 to 0.99). The number needed to treat in order to prevent one perinatal death was 426. There was no significant difference between groups for perineal trauma (RR 1.09, 95% Cl 0.65 to 1.83), postpartum haemorrhage (RR 1.09, 95% Cl 0.92 to 1.30), length of maternal hospital stay (average mean difference -0.34 days, 95% Cl 1.00 to 0.33), or neonatal trauma (RR 1.18, 95% Cl 0.68 to 2.05). IOL was associated with an increase in operative vaginal births (RR 1.07, 95% Cl 0.99 to 1.16), in particular for IOL at < 41 weeks. Systematic reviews conducted prior to this Cochrane review included the same studies and, with the exception of one study [34], were excluded [12, 78–80]. The additional systematic review included reviewed the RCTs within the Cochrane review [6] and compared outcomes associated with IOL at 41 weeks versus 42 weeks [34]. This review identified four RCTs relevant to these timeframes and concluded that there was insufficient evidence to support IOL at 41 weeks instead of 42 weeks. A recent RCT not included in these systematic reviews compared IOL at 41 weeks with EM until 42 weeks found that IOL was associated with reduced adverse perinatal outcomes (1.7% vs 3.1%, absolute risk difference, -1.4%, 95% CI -2.9 to 0.0), however, this study was underpowered to demonstrate superiority of IOL at 41 weeks [35].

The remaining studies were cohort studies, mostly assessed as of moderate or severe risk of bias. In relation to outcomes associated with IOL at different gestational ages, three included retrospective cohort studies compared outcomes under different policy periods [38, 41, 45]. Bleicher et al. [41] compared outcomes for women who gave birth under a policy of IOL at 42 weeks (n = 968; from 2008–2009) with those who gave birth under a policy of IOL at 41 weeks (n = 962; from 2012–2013). This study found that, both the overall CS rate as well as the CS rate for women who underwent IOL, was lower during the 41 week policy period than during the 42 week policy period (15% vs 19.4%, p = 0.014 and 19% versus 27%, p = 0.007). IOL at 41 weeks was also associated with a significant reduction in 1st and 2nd degree perineal lacerations and neonatal readmission within 30 days of discharge. Similarly, Wolff et al. [45] compared outcomes for women who gave birth under a policy of IOL at 41+2 weeks (2012) (n = 8545) versus those who gave birth under a policy of IOL at 42 weeks (2010) (n = 9713). This study found a significant reduction in the rate of CS between 2010 and 2012 (p = 0.05), and a non-significant reduction in perinatal mortality of 60% (from 10 to 3). There were no significant differences in instrumental birth numbers or perinatal outcomes. Kassab et al. [38] compared CS rates associated with a policy of IOL at 41+3 days (n = 124; August 2006 and March 2007) versus a policy of IOL at 42 weeks (n = 227; April 2007 and July 2008), and found that the earlier IOL policy was associated with lower CS rates (p = 0.04) for nulliparous women.

Two cohort studies compared CS rates associated with IOL prior to 41 weeks versus IOL at 41 weeks [36, 47], also presenting opposing findings. A prospective cohort study by Haq et al. [36] compared CS rates for IOL at 40 weeks (n = 39) with IOL at 41 weeks (n = 39), and found that IOL at 41 weeks was associated with lower CS rates (16% vs 29%, p < 0.0001). Conversely, McCoy et al. [47] conducted a secondary analysis of a prospective cohort study to compare CS rates for IOL at term (between 37–40+6) (n = 700) with IOL at >41 weeks (n = 154) among women with an unfavourable cervix (Bishop score of <6 and cervical dilation <2 cm). This study found an increased risk of CS for women induced at >41 weeks versus those induced at term (46.8% vs 26.0%, p < .001).

The remaining seven papers included cohort studies that compared IOL with expectant management (EM) (spontaneous onset of labour or IOL if it becomes indicated) [37, 39, 40, 42–44, 46]. Mya et al. [46] conducted a secondary analysis of two multi-country surveys conducted by the World Health Organization (WHO) on maternal and newborn health to compare outcomes of IOL at 41 weeks (n = 4332) with EM (n = 26,720) (spontaneous onset of labour or birth at > 41 weeks). Compared to IOL, EM was significantly associated with decreased risk of CS in both databases (OR 0.70 and IOR 0.67). A retrospective cohort study by Mahomed et al. [37] compared CS rates associated with IOL at between 40 and 40+6 weeks (n = 2153) versus spontaneous birth between 41 and 41+6 weeks (n = 5658) for nulliparous women with uncomplicated singleton pregnancy, and found that CS rates were significantly higher in the IOL group (OR 1.52; 21% versus 14.9%).

Four other retrospective cohort studies compared the mode of birth associated with IOL at 41+1 weeks to EM until spontaneous onset of labour or IOL at 42–42+6 weeks [40, 42–44]. While, based on a relatively small sample size of 483 women (n = 211 in the IOL group; n = 277 in the EM group), Daskalakis et al. [40] found no significant differences in CS rate (36.5% vs 34.4%) or operative vaginal births (11.4% vs 9.2%) between the two groups, the other studies found that IOL was associated with increased risk of CS. A study by Thangarajah et al. [44], included 856 women (n = 400 in the IOL group; n = 456 in the EM group) and found that IOL was associated with increased rates of CS (33.8% vs. 21.1%, p < 0.001), and perineal lacerations (38.1% vs 26.4%, p = 0.002). Similarly, a study by Pavicic [42] including 1367 women (n = 722 in the IOL group; n = 645 in the EM group) found that IOL was associated with increased CS rates (25.4% vs 16.6%, p = 0.001) and a study by Teo [43] including 6501 women (n = 3588 in the IOL group; n = 2913 in the EM group) found that IOL was associated with higher rates of CS (29.4% vs 18.5%, p = 0.001) and instrumental birth (20.2% vs 17.7%, p = 0.012). The two latter studies were rated as having serious risk of bias as the IOL groups included a significantly higher proportion of women with comorbidities, which were not controlled for.

Lastly, Hermus et al. [39] conducted a retrospective matched cohort study (1:1 ratios for both age and parity) to compare outcomes for women who underwent IOL (= 377) at 42 weeks to EM beyond 42 weeks (n = 377). This study found that EM was associated with non-significantly lower rate of CS (12.5% vs 13.6%, RR 0.9, 95% CI 0.6 to 1.4), but higher incidence of shoulder dystocia (RR 4.3, 95% CI 1.3 to 15) and meconium-stained amniotic fluid (RR 1.8, 95% CI 1.4 to 2.3).

#### Summary statement

Women having IOL beyond 41–42 weeks is associated with fewer perinatal deaths and reduced CS rates, even though the number needed to treat to prevent perinatal mortality is high (approx. 450).

### Premature rupture of membranes (PROM)

Five studies in relation to PROM were included, consisting of two Cochrane reviews [81, 82], one prospective cohort study [83], and two retrospective cohort studies [84, 85]. One Cochrane review and three studies addressed PROM at term (37–42 weeks) [81, 83–85], whilst the other Cochrane review addressed preterm PROM (<37 weeks) [82].

In relation to preterm PROM (< 37 weeks), the Cochrane review by Bond et al. [82] compared outcomes associated with women undergoing a planned early birth (by IOL or CS) with EM between 24 and 37 weeks. The review identified 12 RCTs, with 3617 women [7, 86–96]. In terms of neonatal outcomes, this review identified no clear differences in neonatal sepsis (RR 0.93), neonatal infection (RR 1.24, 95% CI 0.66 to 1.30), and overall perinatal mortality (RR 1.76, 95% CI 0.89 to 3.50), but found that early birth was associated with a higher rate of neonatal death (RR 2.55, 95% CI 1.17 to 5.56), respiratory distress syndrome (RR 1.26, 5% CI 1.05 to 1.53), need for ventilation (RR 1.27, 95% CI 1.02 to 1.58), and NICU admission (RR 1.16, 95% CI 1.08 to 1.24). In terms of maternal outcomes, early birth was associated with an increased rate of CS (RR 1.26, 95% CI 1.11 to 1.44) and endometritis (RR 1.61, 95% CI 1.00 to 2.59), and reduced rate of chorioamnionitis (RR 0.50, 95% CI 0.26 to 0.95). This review conducted a subgroup analysis by gestational age, and compared outcomes associated with planned birth <34 weeks and >34 weeks. The test for subgroup differences were not significant for neonatal infection, respiratory distress syndrome, CS, and chorioamnionitis. There was a decrease in endometritis in women randomised to early birth after 34 weeks [82]. The included studies were at low or unclear risk of bias, with the overall quality of evidence rated as moderate to high.

In relation to PROM at term (≥37 weeks), the Cochrane review by Middleton et al. [81] compared planned early birth (immediate IOL or within 24 hours) with EM (no planned intervention within 24 hours). The review identified 23 RCTs, involving 8615 women [97–117]. Early birth was associated with a reduced risk of maternal infectious morbidity (chorioamnionitis and/or endometritis) (RR 0.49, 95% CI 0.33 to 0.72), and neonates were less likely to have early-onset neonatal sepsis (RR 0.73, 95% CI 0.58 to 0.92). No clear differences were seen in CS rates (RR 0.84, 95% CI 0.69 to 1.04; 23); serious maternal morbidity or mortality (no events); definite early-onset neonatal sepsis (RR 0.57, 95% CI 0.24 to 1.33); or perinatal mortality (RR 0.47, 95% CI 0.13 to 1.66). The quality of included studies was low; only three of the included RCTs were of low risk of bias, while the remaining were of unclear or high risk of bias.

The remaining studies were cohort studies, assessed as of low to moderate risk of bias. A prospective cohort study by Omole-Ohonsi et al. [83] compared immediate IOL (N = 100) with delayed IOL after EM of 12 hours (N = 100). One-third in the delayed IOL group went into spontaneous labour before IOL. Immediate IOL was associated with significantly lower rates of CS (OR 0.18, 95% CI 0.07 to 0.47), operative vaginal birth (OR 0.26, 95% CI 0.07 to 0.88), and higher rates of vaginal birth (OR 6.10, 95% CI 2.76 to 13.75). There was no significant difference in neonatal outcomes.

A retrospective cohort study by Sadeh-Mestechkin et al. [84] compared immediate IOL (N = 213) versus delayed IOL after EM for 48 hours (N = 112). The delayed group had significantly higher rate of prolonged hospitalisation (p = 0.043) (as an indicator for maternal complications), and higher rates of CS (16.4% vs 7.1%, p = 0.024). There was no significant difference in the rate of clinical chorioamnionitis or postpartum endometritis, and there were no cases of early neonatal sepsis. A retrospective cohort study by Pintucci et al. [85] analysed outcomes associated with a policy of delayed IOL after EM for 48 hours (N = 1315). In total, 84% of the women went into spontaneous labour within 48 hours. There were low rates of clinical chorioamnionitis (2.3%) and neonatal infection rate (2.8%). The overall CS rate was 4.5%, which was lower for women who went into labour spontaneously than those who underwent IOL (OR 1.76, 95% CI 1.03 to 3.02).

#### Summary statement

Early birth for **PROM at term** may help reduce maternal and neonatal infections without increasing CS rates.

Early birth for **pre-term PROM** increases the risk of infant death after birth, respiratory problems and NICU admissions, and CS rates, and is associated with a decreased incidence of chorioamnionitis.

### Hypertension/preeclampsia

Fifteen studies in relation to preeclampsia, chronic or gestational hypertension were included. There were three Cochrane reviews [118–120], two RCTs [121, 122], a post hoc analysis of an RCT [123], three prospective cohort studies [124–126], four retrospective cohort studies [127–130], and two case control studies [131, 132]. A further systematic review was excluded as it did not identify any additional studies not already included [133]. The majority of studies focussed on severe preeclampsia as an indicator for IOL, with most of these in women giving birth <34 weeks gestation [119–121, 124–127, 130–132]. One study pertained to gestational hypertension [129], one to chronic hypertension [128], one to late preterm preeclampsia (34–36+6 weeks gestation) [122] and two studies included multiple hypertensive disorders as one group [118, 123].

In relation to hypertensive disorders broadly, Cluver et al. [118] conducted a Cochrane review comparing planned early birth versus EM from 34 weeks to term. The review identified five RCTs, with a total of 1819 women [134–138]. Both the HYPITAT-I (Hypertension and Preeclampsia Intervention Trial at Term) and HYPITAT-II trials were included [134, 135]. The HYPITAT-I trial compared IOL at 36–41 weeks (within 24 hours of randomisation) to EM until spontaneous onset of labour for pregnant women with mild to moderate gestational hypertension or mild preeclampsia [135]. The HYPITAT-II trial compared IOL at 34–36+6 weeks to EM until 37 weeks for pregnant women with gestational hypertension, mild preeclampsia, or deteriorating chronic hypertension [134]. Three further RCTs compared planned early birth via IOL or CS versus EM for pregnant women with mild preeclampsia or gestational hypertension [136, 138] or moderate essential chronic hypertension [137]. The review found a lower risk of composite maternal mortality and severe morbidity for women randomised to receive planned early birth. There was not enough information to draw conclusions about the effects on composite infant mortality and severe morbidity, with contrasting findings in HYPITAT-I (IOL from 36 weeks with RR 0.75, 95% CI 0.46 to 1.28 for composite infant outcome) [135] versus HYPITAT- II (IOL from 34 to 36+6 weeks, RR 1.4 95% CI 1.1 to 1.7 for any neonatal morbidity) [134]. Planned early birth was associated with higher levels of respiratory distress syndrome (RR 2.24, 95% CI 1.20 to 4.18), and NICU admissions (RR 1.65, 95% CI 1.13 to 2.40), with this finding driven by higher neonatal risks in the earlier planned birth group of HYPITAT II [134]. There was no clear difference between the groups for CS or length of hospital stay.

Tajik et al. [123] conducted a post hoc analysis of the HYPITAT-I trial to assess whether cervical ripeness should play a role in the decision for IOL for women with gestational hypertension or mild preeclampsia at term. This trial included a total of 756 women, with 377 in the IOL group, and 379 in the EM group (spontaneous labour). This study found that the superiority of IOL varied significantly according to cervical favourability. The length of the woman’s cervix was a predictor of outcome. For women who were managed expectantly, the longer the cervix, the higher the risk of maternal complications, whereas in women who were induced, cervical length was not associated with increased maternal complications. Similarly, IOL was more likely to reduce the CS rate in women with an unfavourable cervix.

Finally, two retrospective cohort studies sought to determine the optimal timing of birth for women with gestational hypertension [129] and chronic hypertension [128]. Both studies found that IOL between 38- and 39 weeks balances the lowest maternal and neonatal morbidity/mortality for both women with gestational hypertension [129] and those with chronic hypertension [128].

In relation to severe preeclampsia, the majority of included studies assessed outcomes remote from term, i.e. during the very preterm period of less than 34 weeks. Only one study focussed on late preterm preeclampsia (34–36+6 weeks gestation) [122]. An RCT by Chappell et al. (2019) compared planned birth (usually IOL) (n = 448) versus EM (n = 338) in women with late preterm pre-eclampsia from 34 to 37 weeks (The PHOENIX trial) [122]. This study found that planned birth reduced maternal morbidity and severe hypertension (65% vs 75%, RR = 0.86, 95% CI 0·79–0·94; p = 0·0005), but resulted in more neonatal admissions for prematurity (42% vs 34%, RR 1·26, 1·08–1·47; p = 0·0034).

In relation to preeclampsia remote from terms (<34 weeks), a Cochrane review by Churchill et al. [119] compared planned early birth versus EM for severe preeclampsia between 24–34 weeks’ gestation. The review included four RCTs, with a total of 425 women [139–142]. The study found that an expectant approach may be associated with decreased morbidity for the baby. Babies whose mothers were allocated to the early birth group had more intraventricular haemorrhage (RR 1.82, 95%CI 1.06 to 3.14), more hyaline membrane disease (RR 2.30, 95% CI 1.39 to 3.81), required more ventilation (RR 1.50, 95% CI 1.11 to 2.02), were more likely to have a lower gestation at birth, more likely to be admitted to neonatal intensive care (RR 1.35, 95% CI 1.16 to 1.58) and have a longer stay in the NICU, but less likely to be small-for-gestational age (RR 0.30, 95% CI 0.14 to 0.65). There was insufficient data for reliable conclusions on most outcomes for the mother, except that women allocated to the early birth group were more likely to have a CS (RR 1.09, 95% CI 1.01 to 1.18). There was also a second systematic review and meta-analysis of RCTs by Wang et al. [143] which largely included the same studies as the Cochrane review [119], with an additional two studies: the MEXPRE trial [121] and a trial by Duvekot et al. [144]. The latter was closed after 24 months because of low recruitment and the findings were reported by abstract only, and as such is excluded from our review. As the only additional study identified by this review is the MEXPRE trial [121], we have excluded the review by Wang et al. [143] and we report on the MEXPRE trial here.

The MEXPRE trial included 267 women and sought to determine whether EM in women with severe preeclampsia prior to 34 weeks results in improved neonatal outcome in countries with limited resources (i.e. low-middle income countries in Latin America) [121]. This study found no difference in the rate of perinatal mortality (9.4% vs 8.7%; RR 0.91, 95% CI 0.34 to 1.93), composite of neonatal morbidities (56.4% vs 55.6%; RR 1.01, 95% CI 0.81 to 1.26), or maternal morbidity (25.2% vs 20.3%; RR 1.24, 95% CI 0.79 to 1.94) with EM versus early birth. Small for gestational age (21.7% vs 9.4%; RR 2.27, 95% CI 1.21 to 4.14) and placental abruption were more common with EM (RR 5.07, 95% CI 1.13 to 22.7).

Four cohort studies [125–127, 130] and two case control studies [131, 132] assessed the association between mode of birth and maternal and neonatal outcomes for severe preeclampsia remote from term. A prospective cohort study by Ertekin [125] that compared EM (n = 33) versus early birth by IOL or CS (n = 37) for severe preterm preeclampsia on a range of maternal and neonatal outcomes, including the first year of neurological development of infant, did not find statistically significant differences between the two groups. However, there were seven fetal deaths in the immediate birth group versus two in the EM group (P = 0.058). This was most likely due to women with more significant risk factors (e.g. HELLP syndrome) being assigned to the immediate birth group.

The remaining studies addressed the safety of women undergoing an IOL versus a CS for severe preeclampsia remote from term. With the exception of one study [126], these concluded that IOL is a reasonable option that was not associated with poorer maternal and neonatal outcomes [127, 130–132]. A retrospective cohort study by Alanis at al. [127] assessed differences in neonatal outcomes with IOL (n = 282) versus planned CS (n = 209) in women with early onset severe preeclampsia and found that IOL was not associated with an increase in neonatal morbidity or mortality even after controlling for gestational age and other confounders. Similarly, Alexander et al. [130] conducted a retrospective cohort study comparing IOL (n = 145) with planned CS (n = 133) on neonatal outcomes in women whose pregnancies were complicated by preterm severe preeclampsia and birth of very low birth-weight infants. This study found that IOL in cases of severe preeclampsia was not harmful to very low birth-weight infants. While Apgar scores of ≤3 at 5 minutes were more likely in the IOL group (6% versus 2%, P = 0.04), other neonatal outcomes, including respiratory distress syndrome, grade 3 or 4 intraventricular haemorrhage, sepsis, seizures, and neonatal death, were similar in the two groups. Vaginal birth was accomplished by 50 (34%) women in the IOL group.

The two included case control studies had similar findings, and also indicated that IOL for severe preeclampsia should be considered as a reasonable option remote from term rather than a CS [131, 132]. Both studies conducted retrospective chart reviews to determine the rate of vaginal birth after IOL in severe preeclampsia remote from term to identify factors associated with its success and evaluate neonatal outcomes based on induction outcome. Based on a sample of 306 women, Nassar et al. [132] found that of the women that were induced, 48% gave birth vaginally. The Bishop score on admission was the best predictor of success, although the chance of successful IOL increased with advancing gestational age (ranging from 31.6% at </ = 28 weeks' gestation to 62.5% at >32 weeks' gestation). Based on a sample of 250 women, Blackwell et al. [131] found that attempted IOL did not increase neonatal morbidity, and that IOL success was significantly associated with gestational age (rarely successful at <28 weeks). The only study that presented different findings was a prospective cohort study by Mashiloane et al. [126] that compared outcomes associated with planned CS (n = 68), CS following attempted IOL (n = 14), and vaginal birth following successful IOL (n = 26) for severe preeclampsia from 26–32 weeks. This study found that perinatal mortality was significantly higher following IOL (p = 0.0004), and that planned CS contributed to a better perinatal outcome than vaginal birth.

We also identified two other studies in relation to preeclampsia in broader terms, not specified as remote from term. A prospective cohort study by Amorim et al. [124] evaluated the association between mode of birth (vaginal versus CS) and maternal outcomes for 500 women with severe preeclampsia. This study found that the risk of severe maternal morbidity was significantly greater in women in the CS group (54.0% versus 32.7%) irrespective of the presence of labour. Severe maternal morbidity was found to be associated with CS (OR 1.91). Amorim et al. [120] also conducted a Cochrane review to compare maternal and neonatal outcomes for women with severe preeclampsia who had a planned CS versus planned vaginal birth. However, this review did not identify any studies for inclusion and had no results to report.

#### Summary statement

RCT evidence suggests decreased maternal morbidity after IOL for preeclampsia from 34 weeks gestation, however at the cost of increased neonatal morbidity if undertaken at 34–37 weeks. There is little agreement on the timing of birth for women with chronic hypertension or gestational hypertension at term, but there is some evidence that indicates that planned birth between 38 and 39 weeks is associated with the lowest maternal and neonatal morbidity/mortality. EM for severe preeclampsia remote from term increases birthweight and reduces neonatal morbidity.

### Diabetes

Nine studies in relation to diabetes were included, consisting of two Cochrane reviews [9, 145], a secondary analysis of a trial that compared different treatments for mild gestational diabetes mellitus (GDM) [146] and six retrospective cohort studies [147–152]. With the exception of one study [145], all evaluated women with GDM, and excluded those with type I or II diabetes.

The Cochrane review by Bouvain et al. [145] included one RCT that compared outcomes for IOL ≥38 weeks (n = 100) versus EM (until 42 weeks) (n = 100) for pregnant women with diabetes (either type I or type II, or GDM) treated with insulin [153]. Of the 200 participants, 187 women had GDM and 13 had type 2 diabetes. This study found no significant difference between the two groups in terms of CS (RR 0.81, 95% CI 0.52 to 1.26). The risk of macrosomia was reduced in the IOL group (RR 0.56, 95% CI 0.32 to 0.98) and three cases of mild shoulder dystocia were reported in the EM group. No other types of perinatal morbidity were reported.

The Cochrane review by Biesty et al. [9] assessed the effect of planned birth for women with gestational diabetes and included one RCT, the GINEXMAL trial [154]. This trial included 425 women with GDM at term, randomised to IOL (between 38 and 39 weeks) (n = 214) or EM (n = 211) (until 41 weeks). This study found no difference between the two groups in terms of CS rates (RR 1.06, 95% CI 0.64 to 1.77; 12.6% in the IOL group versus 11.7% in the expectant group), or other maternal or neonatal outcomes. There were no maternal or fetal deaths. The study was underpowered and any reported differences between the two groups were very small and not clinically relevant.

There were four retrospective cohort studies that compared outcomes for women with GDM who underwent IOL versus EM, with CS rates as a primary outcome [147, 149–151]. A retrospective cohort study by Bettikher et al. [147] that compared outcomes for IOL (gestation not stated) in 43 women with EM until spontaneous labour in 188 women, found no significant difference between the two groups in terms of CS rate, or any of the other maternal or neonatal outcomes assessed (i.e. the frequency of uterine inertia, uncoordinated contractions and fetal distress). This study concluded that, in the absence of signs of fetal distress or macrosomia, planned early birth was not indicated. Similarly, Grabowska et al. [149] compared CS rates for 96 women who underwent IOL with 32 women who had a spontaneous labour and found that IOL did not increase the risk of CS (25% versus 25%, p = 0.66). A cohort study by Melamed [150] compared outcomes associated with IOL at 38 or 39 weeks and EM for 6417 women with GDM, and found that IOL at 38 or 39 weeks was associated with a lower CS rates but higher risk of NICU admission, when done at <39 weeks gestation.

Four studies assessed the impact of different timings of IOL for women with GDM in terms of CS rates [146, 148] or maternal and neonatal outcomes [151, 152]. A retrospective cohort study by Hochberg et al. (2019) compared outcomes for IOL at 37+0 and 38 + 6 weeks (n = 193) versus 39+0 and 40+6 weeks (n = 237) and found that the rates of composite maternal outcome and composite neonatal outcome did not differ between groups [152]. A retrospective cohort study by Vitner et al. (2019) found that IOL was associated with increased risk for adverse composite neonatal outcome or NICU admission when done prior to 39 weeks [151].

Specific to CS rates, Sutton et al. [146] conducted a secondary analysis of a trial investigating different treatments for mild GDM [155] to compare the rates of CS associated with IOL (n = 220) versus EM (n = 459) at different gestational ages. This study found that IOL was not associated with increased rates of CS at 37, 38, or 39 weeks, but was associated with a 3-fold increase in CS rates at 40 weeks and beyond. A retrospective cohort study by Feghali et al. [148] compared CS rates in women undergoing IOL at each week of gestation, with EM to a later gestational age. Similarly, IOL at 37 weeks, 38 weeks and 39 weeks was associated with similar rates of CS than EM, particularly for nulliparous women. The difference in CS rates between the two groups was only significant at 38 weeks for multiparous women. This study did not report on outcomes at 40+ weeks. The included cohort studies were all rated as having a moderate to serious risk of bias, as the reason for IOL was not clearly defined, therefore findings need to be interpreted with caution.

#### Summary statement

There was little quality evidence to inform management between IOL at term or EM for women with diabetes during pregnancy, and the little evidence that was available was largely limited to GDM. Only one relevant study included women with pre-existing diabetes (Type I and Type 2), consisting of only 13 women.

### Twin pregnancy

We identified five studies in relation to twin pregnancy, consisting of a Cochrane review [156], and four retrospective cohort studies [157–160]. None of the included studies conducted an analysis by chorionicity. Chorionicity data was unavailable or incomplete in three studies [156, 158, 159], and two studies had the data but did not perform this sub-analysis [157, 160].

The Cochrane review [156] compared elective birth from 37 weeks to EM for women with an otherwise uncomplicated twin pregnancy. This review identified two RCTs for inclusion, involving a total of 271 women and 542 infants [156, 161]. Women in the elective birth group (n = 133) had a planned birth at 37 weeks by either CS or IOL in one of the included studies [162], and by IOL in the other study [161]. Women randomised to the EM group (n = 138) had their care according to local hospital guidelines, which involved either awaiting the spontaneous onset of labour, or having a planned birth after 38 weeks. The review found no statistically significant differences in risk of CS, perinatal death or serious infant morbidity, or maternal death or serious maternal morbidity, although both perinatal (RR 0.34, 95%CI 0.01 to 8.35) and maternal composite morbidity and mortality (RR 0.29, 95% CI 0.06 to 1.38) were lower (albeit not statistically) in the elective birth group.

A retrospective cohort study by Tavares et al. [160] compared outcomes for IOL versus spontaneous vaginal birth in twin pregnancy after 36 weeks of gestation. Of the 288 women with multiple pregnancies during the study period, 75 twin pregnancies >36 weeks of gestation were included, with 33 women undergoing IOL and 42 women who went into labour spontaneously. This study found no statistical differences between the two groups in terms of maternal and neonatal morbidity, or admission to the NICU, but did find a significant increase in CS in the IOL group (60.6 vs. 33.3%, p<0.05).

The remaining cohort studies examined birth outcomes in twin pregnancies. A study by de Castro et al. [158] measured twin pregnancy labour outcomes, with a subgroup comparison for women who underwent IOL (n = 287) with women who had a spontaneous vaginal birth for both twins, a CS or a vaginal birth for first twin and CS for 2nd twin (n = 596). This study found that IOL significantly reduced the chance of achieving vaginal birth (Foley catheter induction 74.7%; non-Foley induction 86.7%; no induction 88.9%, p < 0.001). Similarly, Jonsson [157] retrospectively compared outcomes for women who underwent IOL (n = 220) with those who had a spontaneous labour (n = 242), and found that IOL in twin pregnancies increased the risk of CS compared with spontaneous labour onset, especially if Foley catheters or prostaglandins were used. However, approximately 80% of babies born from women who had an IOL were born vaginally. These findings are not supported by Hamou et al. [159] which compared outcomes for women who had an IOL (n = 653); spontaneous birth (n = 2937) versus elective CS (n = 1015). While consistently this study found that the rate of vaginal birth in the IOL group was 81%, IOL was found to be associated with a lower rate of CS than spontaneous labour (77% reduction, OR 0.23) and lower rates of neonatal death (78% reduction, OR 0.22). However, this study was assessed as of serious risk of bias, and the high adverse outcome observed in the spontaneous twin group is likely because a large proportion of them (25%) were early preterm, prior to 34 weeks.

#### Summary statement

While some cohort studies found that IOL in twin pregnancies increases the risk of CS compared to spontaneous labour onset, other studies found the reverse. Evidence from two RCTs (included in a Cochrane review) found non-significant improvements in composite neonatal and maternal outcomes with planned birth for twins at 37 week.

### Intrahepatic cholestasis of pregnancy

In relation to intrahepatic cholestasis of pregnancy (ICP), we identified three studies, consisting of a Cochrane review [163] and two retrospective cohort studies [164, 165]. The Cochrane review [163] evaluated the effectiveness and safety of interventions in women with cholestasis of pregnancy. While this review identified 21 RCTs, only one included RCT, the PITCH trial, compared outcomes for early term birth to EM (and as such is the only RCT relevant here) [166]. The PITCH trial included 63 women, 30 of which were randomised to IOL between 37–37+6 weeks and 33 randomised to EM (spontaneous labour until 40 weeks or CS undertaken by normal obstetric guidelines, usually after 39 weeks). There were no stillbirths or neonatal deaths in either group and no significant differences in CS (RR 0.68), passage of meconium-stained liquor (RR 0.55, 95% CI 0.15 to 2.01) or admission to NICU (RR 0.55, 95% CI 0.05 to 5.76). This study was underpowered to detect other clinically important differences. Subgroup analysis results by bile acid level were not reported.

The retrospective cohort study by Kohari et al. [165] sought to determine the efficacy of a planned early birth policy for women with severe ICP (bile acids >40μmol/L) by comparing outcomes for women who gave birth under an active management policy (between 2009–2013) to those who were cared for prior to the introduction of this policy (between 2005–2008). Women with ICP who gave birth under the active management policy (n = 128) were managed as inpatients and had a planned birth between 36 and 37 weeks. Prior to the introduction of this policy, decisions around mode and timing of birth were made at the discretion of the health professional. The active management policy was found to be associated with a significant reduction in the incidence of stillbirth (0% versus 3.4%, p = 0.035). There was no difference in CS rates or NICU admissions. Women’s demographic characteristics were similar between the groups, with the exception of greater maternal age and GDM in the newer cohort. A retrospective cohort study by Puljic et al. [164] sought to determine the optimal timing of birth for pregnancies complicated by ICP (no stratification by bile acid level), by comparing outcomes by each additional week (from 34–40 weeks) of EM versus immediate birth. This study found that birth at 36 weeks gestation was associated with lower perinatal mortality.

#### Summary statement

The evidence is mixed. One RCT found that early planned birth for ICP was not associated with improved outcomes, however this study was underpowered to detect clinically important differences. Evidence from retrospective cohort studies suggests that planned early birth was associated with a significant reduction in the incidence of stillbirths, and that planned birth at 36 weeks gestation was associated with lower perinatal mortality.

### Elevated maternal body mass index

In relation to elevated maternal BMI (≥30.0 kg/m2), four retrospective cohort studies were included [167–170], presenting mixed findings. Wolfe et al. [170] compared maternal and neonatal outcomes in obese (≥30.0 kg/m2), nulliparous women with an unfavourable cervix (modified Bishop score < 5) undergoing elective IOL between 39 and 41 weeks (n = 60) with EM after 39 weeks (n = 410). This study found that IOL was associated with higher rates of CS (40% vs 25.9%, p = .022), and NICU admissions (18.3% vs 6.3%, p = .001). Other maternal and neonatal outcomes were similar. These findings were not supported by the other included studies, described below, which found that IOL was associated with lower CS rates [167–169].

Kawakita et al. [167] compared the CS rate of elective IOL with EM in morbidly obese women (BMI ≥40 kg/m2) between 37 and 41+6 weeks who had singleton pregnancies, cephalic presentations and no previous CS or other comorbidity. In nulliparous women, elective IOL was not associated with increased risks of CS and was associated with decreased risks of macrosomia (2.2% vs 11.0%) at early term and decreased NICU admissions (5.1% vs 8.9%) at full term. In multiparous women, IOL was associated with a decreased risk of macrosomia at early term (4.2% vs 14.3%), CS at full term (5.4% vs 7.9%), and composite neonatal outcome (0% vs 0.6%) at full term.

Both Lee et al. [169] and Pickens et al. [168] compared outcomes between elective IOL and EM in obese women (BMI ≥30.0 kg/m2) with singleton pregnancies by analysing a Californian national dataset. Lee et al. [169] analysed 2007 data and compared outcomes for the two groups for each gestational age, from 37–40 weeks. This study found that IOL was associated with lower CS rates, and lower odds of macrosomia. There were no differences in the other reported outcomes. Similarly, Pickens et al. [168] analysed data from 2007 to 2011, comparing outcomes for these two groups at 39 and 40 and 41 weeks. This study found that IOL was associated with reduced CS rates (at 39 weeks gestation, frequencies were 35.9% vs 41.0%, p = <0.05), reduced composite of severe maternal morbidity (5.6% vs 7.6%, p = <0.05), and reduced NICU admissions (7.9% vs 10.1%, p = <0.05).

#### Summary statement

The evidence is mixed and from retrospective cohort studies only. While some studies indicated that IOL for a high BMI was associated with reduced CS rates and improved maternal and neonatal outcomes, other studies demonstrated the reverse.

### Maternal age

In relation to maternal age, we found two studies to include, one RCT [171] and one retrospective cohort study [172]. An RCT by Walker et al. [171] tested if IOL at 39 weeks reduces CS rates for nulliparous women who are ≥ 35 years by comparing outcomes for IOL (n = 305) with EM (n = 314). This study found no significant differences in the two groups in terms of CS rates (32% in the IOL group vs 33% in the EM group; RR 0.99, 95% CI 0.87 to 1.14), the percentage of women who had a vaginal birth with the use of forceps or vacuum (38% vs 33%, RR 1.30, 95% CI 0.96 to 1.77), the women’s experience of childbirth, or adverse maternal or neonatal outcomes. There were no maternal or infant deaths.

A retrospective cohort study by Knight et al. [172] compared perinatal mortality between IOL (at between 39 and 41 weeks) (n = 25,583) and EM (n = 51,744) for nulliparous women aged ≥ 35 years. Women with comorbidities or previous complicated births were excluded. IOL at 40 weeks was associated with a lower risk of in-hospital perinatal death (0.08% vs 0.26%; RR 0.33 95% CI 0.13 to 0.80; p = 0.015) and meconium aspiration syndrome (0.44% vs 0.86%; RR 0.52, 95% CI 0.35 to 0.78; p = 0.002), but an increased risk of instrumental vaginal birth (RR 1.06, 95% CI 1.01 to 1.11; p = 0.020) and CS (RR 1.05, 95% CI 1.01 to 1.09; p = 0.019). A number needed to treat analysis indicated that 562 women would require IOL at 40 weeks to prevent one perinatal death.

#### Summary statement

Evidence from one RCT indicated that IOL does not improve outcomes or CS rates for women greater than 35 years, however this study was underpowered to identify the effect of IOL on perinatal death. Evidence from a retrospective cohort study suggested that IOL at 40 weeks reduces perinatal mortality.

### Maternal cardiac disease

One study, a prospective cohort study, addressed maternal cardiac disease [173]. This study examined the safety of IOL in women with cardiac disease by comparing outcomes for women who underwent IOL between 37 and 40 weeks (n = 47) versus EM (n = 74) (spontaneous onset of labour resulting in vaginal birth or emergency CS). There was no significant difference in complication rate between the two groups, however the groups were not well matched as women in the IOL group had more severe cardiac disease than those in the EM group.

### Suspected macrosomia

In relation to suspected macrosomia as an indication for women undergoing an IOL, there were three studies, consisting of a Cochrane review [174], a systematic review and meta-analysis of RCTs and observational studies [175] and one retrospective cohort study [176]. We also identified a fourth study, a systematic review and meta-analysis, but this review included the same papers as the Cochrane review (the Cochrane was assessed as higher quality) and was therefore excluded [177].

The Cochrane review by Boulvain (2016) [174] sought to determine the effects of a policy of IOL of between 37 and 40 weeks for suspected fetal macrosomia on CS rates and maternal or perinatal morbidity. The review identified four RCTs [178–181] with a total of 1190 women, 590 in the IOL group and 600 in the EM group. In two of the included trials, women with diabetes were excluded [179, 180], one trial excluded women treated with insulin, but included participants who had GDM controlled by diet (10%) [178], whilst the participant inclusion criteria for the fourth trial was unclear [181]. Women were included when the fetal weight, estimated by ultrasound examination, was between 4000g and 4500g [180], and between 4000g and 4750g [179], when the fetus was estimated to weigh >97th percentile [181], or >95th centile [178]. This review found that compared to EM, IOL had no clear effect on the risk of CS (RR 0.91, 95% CI 0.76 to 1.09) or instrumental birth (RR 0.86, 95% CI 0.65 to 1.13) but did reduce shoulder dystocia (RR 0.60, 95% CI 0.37 to 0.98) and fetal fracture (any) (RR 0.20, 95% CI 0.05 to 0.79). There was no strong evidence of any difference between groups for low infant Apgar scores (<7 at one minute) (RR 1.51, 95% CI 0.25 to 9.02) or low arterial cord blood pH (RR 1.01, 95% CI 0.46 to 2.22). There were no clear differences between groups for brachial plexus injury, although this outcome was infrequent. Two studies reported third- and fourth-degree perineal tears, but only one had estimable data [178]; this study found the number of women with perineal tears was increased in the IOL group (RR 3.70, 95% CI 1.04 to 13.17). There was no perinatal mortality, and no differences in the groups in terms of the number of newborns with intraventricular haemorrhage (RR 1.06, 95% CI 0.19 to 5.96), or neonatal intensive care admissions (RR 0.66, 95% CI 0.35 to 1.24).

The systematic review and meta-analysis by Sanchez-Ramos et al. [175] included two of the RCTs included in the Cochrane review [179, 180], in addition to nine observational studies published between 1966 and 2002, with a total of 3751 women (2700 in the IOL group and 1051 in the EM group). The observational and RCT data were analysed separately; since we have already reported on the RCT findings, we only report the findings of the observational studies here. Analysis of the non-randomised studies indicates that the risk of CS may be increased when IOL is undertaken. Women who experienced spontaneous onset of labour had a lower incidence of CS in comparison to the IOL group (OR 0.39, 95% CI 0.30 to 0.50) and higher rates of spontaneous vaginal birth (OR 2.07, 95% CI 1.34 to 3.19). No differences were noted in rates of operative vaginal births, incidence of shoulder dystocia, or abnormal Apgar scores, in the analyses of the observational studies.

An observational study by Cheng et al. [176] based on known birthweight presented different findings. This retrospective cohort study compared the frequency of CS for women who had an IOL at 39 weeks with a neonatal birthweight of 4000 +/- 125g (birthweight 3875-4125g) with women who gave birth (following IOL or spontaneous onset of labour) at 40 weeks with birthweight 4075–4325g, at 41 weeks with a birthweight at 4275–4525g, or 42 weeks with a birthweight of 4475–4725g (assuming an intrauterine fetal weight gain of 200g per additional week of gestation). The frequency of CS in the IOL group was lower compared with women who gave birth at a later gestational age (35.2% versus 40.9%; OR 1.25, 95% CI 1.17 to 1.33). This study concluded that in the setting of macrosomia and known birthweight, IOL may reduce CS rates.

#### Summary statement

Evidence from four RCTs included in a Cochrane review indicates that there appears to be little difference between IOL versus EM in terms of maternal and neonatal outcomes for women with suspected macrosomia.

### Fetal gastroschisis

In relation to known fetal gastroschisis, we identified two studies for inclusion, a Cochrane review [182] and a retrospective cohort study [183]. The Cochrane review [182] assessed the effects of elective preterm birth (<37 weeks) for fetal gastroschisis, and identified one RCT for inclusion [184]. This RCT assessed whether planned birth at 36 weeks reduces postnatal morbidity without exposing the infant to the added risks of prematurity by comparing outcomes for IOL at 36 weeks (n = 21) and spontaneous onset of labour (n = 21). The trial found no significant benefits or adverse effects associated with elective preterm birth, however, it was underpowered to detect clinically important differences. Two babies died in the planned birth group versus none in the spontaneous group. Seven women (33%) in the planned birth group and nine women (43%) in the spontaneous group had a CS (RR 0.78, 95% CI 0.36 to 1.70). There were no statistical differences in birthweight, ventilation requirements, necrotising enterocolitis and requirements for repeat surgery between the two groups. The average gestational age at birth was 35.8 weeks in the planned birth group and 36.7 weeks in the spontaneous onset of labour group.

An observational study by Al-Kaff et al. [183] analysed data from a national dataset in Canada that included 519 fetuses diagnosed with isolated gastroschisis between 2005 and 2013. This study compared outcomes for mode of birth (spontaneous labour, n = 190; IOL, n = 280; CS, n = 49) and timing of birth (≤35 weeks, n = 8; 36–37 weeks, n = 193; ≥38 weeks, n = 69). Neither mode nor timing of birth were associated with significant benefits or adverse effects. Planned IOL was not associated with decreased length of neonatal stay, total parenteral nutrition duration, or risk of the composite adverse outcome (RR 1.7, 95% CI 0.1 to 3.2) compared with birth following spontaneous onset of labour. Planned birth at 36–37 weeks was not associated with decreased length of neonatal stay, total parenteral nutrition duration or risk of composite outcome (RR 2.3, 95% CI 0.8 to 5.4) compared with planned birth at 38 weeks. Findings support awaiting the onset of spontaneous labour in pregnancies that are complicated by fetal gastroschisis.

#### Summary statement

There was no evidence to support IOL for women who have pregnancies complicated by fetal gastroschisis.

### Suspected fetal compromise

We identified four studies in relation to fetal compromise, consisting of two Cochrane reviews [185, 186] and two retrospective cohort studies [187, 188]. The included studies regarded compromise in the context of oligohydramnios [185, 187] as well as suspected IUGR [185, 186, 188]. The Cochrane review by Stock et al. [186] assessed the effects of immediate versus deferred birth of preterm babies with suspected fetal compromise on neonatal, maternal and long-term outcomes. This review included one trial of 548 women (588 babies) with pregnancies between 24 and 36 weeks and compared the outcomes for immediate birth (by IOL or CS) (n = 296) versus deferred birth (n = 291) (i.e. a set period of time until test results worsen, or until spontaneous onset of labour) [139, 189]. More women in the immediate birth group had a CS (RR 1.15, 95% CI 1.07 to 1.24), there were more babies who were ventilated for more than 24 hours (RR 1.54, 95% CI 1.20 to 1.97), and more had cerebral palsy at or after two years of age (RR 5.88, 95% CI 1.33 to 26.02). There was no real difference for other neonatal morbidity and mortality outcomes, perinatal mortality (RR 1.17, 95% CI 0.67 to 2.04), the composite outcome of death or disability at or after two years of age (RR 1.22, 95% CI 0.85 to 1.75), neurodevelopment impairment at or after two years (RR 1.72, 95% CI 0.86 to 3.41), death at or after two years of age (RR 1.04, 95% CI 0.66 to 1.63), or death or disability in childhood (6–13 years of age) (RR 0.82, 95% CI 0.48 to 1.40). The gestational age at birth was a median of four days earlier in women randomised to immediate birth. The review concluded that for preterm babies with suspected compromise and uncertainty about whether to hasten the birth or not, there appeared to be no benefit to immediate birth.

The Cochrane review by Bond et al. [185] assessed the effects of immediate birth (via IOL or CS) versus EM (until spontaneous onset of labour or planned early birth if it became necessary) for suspected fetal compromise at term (≥ 37 weeks) on neonatal, maternal and long-term outcomes. This review identified three RCTs for inclusion, with a total of 546 participants, of which 269 were randomised to immediate birth and 277 to EM. Two of the trials compared outcomes in 492 women whose pregnancies were complicated by IUGR [190–192], and one trial included 54 women with oligohydramnios [193]. This review found no difference in neonatal outcomes including perinatal mortality (no deaths in either group), major neonatal morbidity (RR 0.15, 95% CI 0.01 to 2.81), or neurodevelopmental disability/impairment at two years of age (RR 2.04, 95% CI 0.62 to 6.69). There was no difference in the risk of necrotising enterocolitis or meconium aspiration. There was also no difference in maternal mortality (RR 3.07, 5% CI 0.13 to 74.87), significant maternal morbidity (RR 0.92, 95% CI 0.38 to 2.22), CS rates (RR 1.02, 95% CI 0.65 to 1.59) or secondary maternal outcomes. Significantly more infants in the planned early birth group were admitted to an intermediate care nursery (RR 1.28, 95% CI 1.02 to 1.61). The gestational age at birth was an average of 10 days earlier in women randomised to immediate birth.

A retrospective cohort study by Rabinovich et al. [188] compared outcomes for IOL (n = 1428) versus EM (n = 804) between 34 and 38 weeks for IUGR. This study found that the IOL group had lower stillbirth and neonatal death rates (p < .001), higher 1 and 5 min Apgar scores and higher vaginal birth rates. IOL at 37 weeks protected from stillbirth but not from adverse composite neonatal outcomes. A retrospective cohort study by Brzezinski-Sinai et al. [187] compared outcomes for women with isolated oligohydramnios between 34 and 36.6 weeks who went into labour spontaneously (n = 33) versus those who underwent IOL (n = 111). Spontaneous labour was associated with statistically significant higher rates of CS (p < .001), as well as higher rates of maternal infection, chorioamnionitis, and transitory tachypnoea of the newborn. This study concluded that IOL may be beneficial to both the neonate and the mother; however some caution needs to be used interpreting these findings as the study was assessed as of moderate risk of bias.

#### Summary statement

For preterm babies with suspected compromise and uncertainty about whether to plan birth early or not, there appears to be no benefit to immediate birth. However, included studies were largely underpowered and had different definitions of what is considered fetal compromise.

## Discussion

The majority of indications for IOL are not supported by strong evidence. While there is high quality evidence in relation to IOL for post-term pregnancy, hypertension/preeclampsia and PROM, for all other indications there were conflicting findings and/or insufficient power to provide definitive evidence. A summary of the evidence and recommendations for future research are included in Table 3.

**Table 3:** Evidence summary and recommendations for future research.

**Post-term pregnancy**
*Evidence*:
IOL versus EM, beyond 41–42 weeks is associated with fewer perinatal deaths and reduced CS rates. Therefore, although the number needed to treat is high for prevention of perinatal mortality, at approximately 450 IOL for every death prevented [6], and rate of assisted vaginal birth increased, post-term pregnancy is likely to remain a routine IOL indication. The available evidence for optimal timing of IOL (41+ versus 42+) remains limited.
*Future directions*:
More studies with an adequate sample size would be ideal to improve the granularity of the data at different gestations. While further research is required, enthusiasm for post-term pregnancy trials may be low after the recently published ARRIVE RCT, where reduction in CS rates (and no evidence of maternal or infant harm) was found in women undergoing IOL at 39+ weeks versus EM [18]. Furthermore, given that offering IOL at 41+ weeks is ingrained into policy and practice in most high-income settings [13, 16, 194] it is likely any sufficiently powered RCT would need to be conducted in a low-to-middle-income country setting. These findings may then not necessarily be applicable to high-income settings.
**PROM at term**
*Evidence*:
Evidence from a Cochrane review that included 23 RCTs indicates that planned early birth may help reduce maternal and neonatal infections without increasing CS rates. The quality of the evidence was low due to many studies being at high risk of bias.
*Future directions*:
Ideally further research to assess the benefits versus harms of planned early birth would be performed, particularly in the early term (37–38 week) group. However, evidence that risk of chorioamnionitis increases from approximately 12 hours after term PROM [195], and associations of chorioamnionitis with the serious long-term morbidity of cerebral palsy, in addition to the short term morbidities assessed in the included studies [196], means there is unlikely to be clinical equipoise to perform such trials.
**Pre-term PROM**
*Evidence*:
Evidence from a Cochrane review that included 12 RCTs indicated that early birth increased the risk of infant death after birth, respiratory problems and NICU admissions, and CS rates without a clinically important difference in the incidence of neonatal sepsis. Early birth was associated with decreased incidence of chorioamnionitis. For women with PROM <37 weeks with no contraindications to continuing the pregnancy, a policy of EM with careful monitoring was associated with better perinatal outcomes.
*Future directions*:
More research that explores the risk benefit ratio of early birth (late preterm) on long term developmental outcomes is required.
**Hypertension**
*Evidence*:
There was limited high quality evidence to inform decisions about optimal timing of birth. There was little or no agreement on the timing of birth for women with chronic hypertension, gestational hypertension or mild preeclampsia at term. Some evidence indicated that planned birth between 38 and 39 weeks was associated with the lowest maternal and neonatal morbidity/mortality for both women with gestational hypertension and those with chronic hypertension. In preeclampsia or gestational hypertension, maternal morbidity was lower in RCTs comparing immediate (versus delayed) birth anytime from 34 weeks, however infant morbidity may be higher, particularly prior to 37 weeks. The vast majority of studies regarded severe preeclampsia remote from term. There was evidence that indicated that EM for severe preeclampsia remote from term increases birthweight and reduces neonatal morbidity. In relation to IOL versus CS, evidence indicated that while IOL is associated with high rates of CS, it is not associated with increased harm and should be considered a reasonable option.
*Future directions*:
Overall the strength of the evidence was weak, particularly regarding preterm preeclampsia and timing of birth at term for chronic hypertension and gestational hypertension, and more research is needed.
**Diabetes**
*Evidence*:
There was little quality evidence to inform management between IOL at term or EM, and the little evidence that was available was limited to GDM. Only one relevant study included women with pre-existing diabetes (Type I and Type 2), consisting of only 13 women.
*Future directions*:
Further prospective cohort and RCT studies are required.
**Twin pregnancies**
*Evidence*:
The existing evidence does not definitively indicate that early planned birth for uncomplicated twin pregnancy improves outcomes. While some cohort studies found that IOL in twin pregnancies increases the risk of CS compared to spontaneous labour onset, other studies found the reverse. Evidence from two RCTs found non-significant improvements in composite neonatal and maternal outcomes with planned birth at 37 weeks.
*Future directions*:
More research is required. However, given population data suggests increased stillbirth risk in twin pregnancy beyond 37 weeks, there is unlikely to be uptake for trials of birth versus EM at later gestations [156]. Complicating interpretation of the twin pregnancy evidence is the non-reporting, missing data, or lack of power of the underlying studies to examine monochorionic (MCDA) versus dichorionic (DCDA) data. MCDA twins have substantially different risks to DCDA, including consequences of late intrauterine death of one twin on the other twin, making grouping of MCDA and DCDA in timing of birth studies problematic [197].
**Intrahepatic cholestasis of pregnancy**
*Evidence*:
Evidence from one RCT indicated that early planned birth was not associated with improved outcomes, however this study was underpowered to detect clinically important differences. Evidence from retrospective cohort studies suggested that planned early birth was associated with a significant reduction in the incidence of stillbirths, and that planned birth at 36 weeks gestation was associated with lower perinatal mortality.
*Future directions*:
Further well-conducted cohort studies or RCTs of early term (37–38 weeks) versus full-term (39–40 weeks) birth are recommended. Timing of birth RCTs may not be ethical in the subgroup of women with high bile acids (>40μmol/L), where there is some cohort data to suggest an increased stillbirth risk directly related to ICP [198, 199], and would certainly be hard to justify in those with bile acid levels >100 μmol/L where cohort evidence for increased stillbirth risk is strong [200]. However for the majority of cholestasis cases, where bile acid levels are 10–40 μmol/L, an elevated stillbirth risk above background is not proven [199, 200], and early term birth with its potential adverse infant neurodevelopmental sequelae may not be justified [201].
**Maternal elevated BMI**
*Evidence*:
Evidence from retrospective cohort studies presented mixed findings. While some studies indicated that IOL was associated with reduced CS rates and improved maternal and neonatal outcomes, other studies demonstrated the reverse.
*Future directions*:
Further prospective cohort studies and RCTs are required.
**Maternal age**
*Evidence*:
Evidence from one RCT indicated that IOL does not improve outcomes or CS rates for women greater than 35 years, however this study was underpowered to identify the effect of IOL on perinatal death. Evidence from a retrospective cohort study suggested that IOL at 40 weeks reduces perinatal mortality.
*Future directions*:
Further research is required.
**Suspected macrosomia**
*Evidence*:
Evidence from four RCTs included in a Cochrane review indicates that there appears to be little difference between IOL versus EM in terms of maternal and neonatal outcomes, but most included studies were underpowered. Evidence from cohort studies present mixed findings, with some indicating that IOL is associated with a reduction in CS rates, and some indicating IOL is associated with increased CS rates.
*Future directions*:
Further adequately powered research is required.
**Fetal gastroschisis**
*Evidence*:
Neither the Cochrane review (with one RCT included) nor the retrospective cohort study found any significant benefits or adverse effects associated with IOL. While the RCT was underpowered, these findings support EM in pregnancies that are complicated by fetal gastroschisis.
*Future directions*:
As more women globally are exposed to antenatal ultrasound, more babies may be identified in utero with fetal gastroschisis. Therefore, further research is necessary to determine the impact of IOL especially in low to middle income settings.
**Fetal compromise**
*Evidence*:
Evidence from RCTs included in Cochrane reviews indicates that for preterm babies with suspected compromise and uncertainty about whether to plan give birth early or not, there appears to be no benefit to immediate birth. However, these studies were largely underpowered.
*Future directions*:
Further adequately powered research is required.

We did not identify any studies for inclusion in relation to fetal alloimmune disease, antepartum haemorrhage, chorioamnionitis (including suspected), or maternal mental health indications, and only one study in relation to maternal cardiac disease, which did not identify any adverse nor beneficial effects associated with IOL. This may be due to the low incidence of these issues and therefore the challenges in undertaking research. Clinical judgement rather than high level evidence will need to continue to drive practice.

The uncertainty in the evidence identified by this review raises questions about the implications for evidence in practice and the development of guidelines. While variations in clinical practice is often attributed to suboptimal guideline adherence [202–204], there is an increasing recognition that this may also be due to a shortage of clear clinical guidelines that provide consistent recommendations that are evidence based [203, 205, 206]. Specific to IOL a recent review of guidelines identified significant variation across guidelines in what are considered acceptable indications for IOL [17]. This variation can be understood in light of the evidence gaps in this space.

While further high quality evidence needs to be developed, there are challenges associated with RCTs, in terms of recruitment and funding. Until this evidence is available, there is a need to develop a better understanding of how to provide evidence-based care when the evidence is unclear. Clinical decision making should be informed by evidence from RCTs and cohort studies, but evolve to include the women’s experience of care and preferences and include a process of shared-decision making. While the importance of shared decision making between women and practitioners is increasingly recognised [207, 208], how shared decision making is best performed in the context of uncertainty or ambiguity remains less clear [209]. To support and inform evidence based care, more research is needed into shared decision making in a context of uncertainty. Furthermore, the experiences and preferences of women is largely absent from this literature, and RCTs that include women’s experience of care as an outcome are required.

This review presents a comprehensive overview of the literature in relation to IOL. However, we could not access the full text of a small number of articles, and we did not include studies that were not in English that may have been relevant to our findings. Furthermore, in accordance with the scoping methodology [29, 210], the quality assessment of the included studies was minimal and largely limited to an assessment of bias. Strengths of this study were that two authors reviewed each of the articles and recognised tools for data extraction and bias detection were used.

## Conclusion

A large proportion of pregnant women have IOL at or near term, sometimes for indications that are well supported by evidence, and sometimes not. This study systematically mapped the available evidence for common indications for IOL. While for some indications, IOL is highly recommended, a number of common indications do not have strong supporting evidence. Overall, few RCTs have evaluated the various indications for IOL, and researchers and funding agencies should prioritise studies of sufficient power that can help to guide care in these situations. However, the entrenched nature of some IOL indications even when not well supported by evidence does present practical difficulties to RCT recruitment. Women should be provided with the best available evidence to help them make an informed choice about the risks and benefits of IOL. Clinicians should use the best available evidence to inform decision making and acknowledge when insufficient evidence is available.

## Supporting information

S1 Checklist Preferred reporting items for systematic reviews and meta-analyses extension for scoping reviews (PRISMA-ScR) checklist. (DOCX)
